# Krill oil improved osteoarthritic knee pain in adults with mild to moderate knee osteoarthritis: a 6-month multicenter, randomized, double-blind, placebo-controlled trial

**DOI:** 10.1093/ajcn/nqac125

**Published:** 2022-07-26

**Authors:** Welma Stonehouse, Bianca Benassi-Evans, Jana Bednarz, Andrew D Vincent, Stephen Hall, Catherine L Hill

**Affiliations:** Health and Biosecurity, Commonwealth Scientific and Industrial Research Organisation (CSIRO), Adelaide, South Australia, Australia; Health and Biosecurity, Commonwealth Scientific and Industrial Research Organisation (CSIRO), Adelaide, South Australia, Australia; Adelaide Health Technology Assessment, School of Public Health, University of Adelaide, Adelaide, South Australia, Australia; Freemasons Centre for Male Health & Wellbeing, School of Medicine, University of Adelaide, Adelaide, South Australia, Australia; Emeritus Research Pty Ltd, Camberwell, Victoria, Australia; Faculty of Medicine, Nursing and Health Sciences, Monash University, Melbourne, Victoria, Australia; Rheumatology Unit, The Queen Elizabeth and Royal Adelaide Hospitals, Adelaide, South Australia, Australia

**Keywords:** osteoarthritis, knee osteoarthritis, knee pain, krill oil, omega-3, eicosapentaenoic acid, docosahexaenoic acid

## Abstract

**Background:**

Osteoarthritis (OA) is a major cause of chronic pain and disability worldwide. Treatment generally focuses on symptom relief through nonsteroidal anti-inflammatory drugs (NSAIDs) and analgesics, which may incur side effects. Krill oil, rich in anti-inflammatory long-chain (LC) omega-3 ( ω–3) PUFAs and astaxanthin, may be a safe and effective alternative treatment.

**Objectives:**

This study sought to investigate the effects of a commercially available krill oil supplement on knee pain in adults with mild to moderate knee OA. Secondary outcomes were knee stiffness; physical function; NSAID use; Omega-3 Index; and lipid, inflammatory, and safety markers.

**Methods:**

Healthy adults (*n* = 235, 40–65 y old, BMI >18.5 to <35 kg/m^2^), clinically diagnosed with mild to moderate knee OA, regular knee pain, and consuming <0.5 g/d LC ω-3 PUFAs, participated in a 6-mo double-blind, randomized, placebo-controlled, multicenter trial. Participants consumed either 4 g krill oil/d (0.60 g EPA/d, 0.28 g DHA/d, 0.45 mg astaxanthin/d) or placebo (mixed vegetable oil). Knee outcomes were assessed using the Western Ontario and McMaster Universities Osteoarthritis Index (WOMAC) numeric scale (normalized to scores of 0–100). Outcomes were assessed at baseline, 3 mo, and 6 mo.

**Results:**

Omega-3 Index increased with the krill oil supplement compared with placebo (from 6.0% to 8.9% compared with from 5.5% to 5.4%, *P* < 0.001). Knee pain score improved in both groups with greater improvements for krill oil than for placebo (difference in adjusted mean change between groups at 6 mo: −5.18; 95% CI: −10.0, −0.32; *P* = 0.04). Knee stiffness and physical function also had greater improvements with krill oil than with placebo (difference in adjusted mean change between groups at 6 mo: −6.45; 95% CI: −12.1, −0.9 and −4.67; 95% CI: −9.26, −0.05, respectively; *P* < 0.05). NSAID use, serum lipids, and inflammatory and safety markers did not differ between groups.

**Conclusions:**

Krill oil was safe to consume and resulted in modest improvements in knee pain, stiffness, and physical function in adults with mild to moderate knee OA.

This trial was registered at clinicaltrials.gov as NCT03483090.

See corresponding editorial on page 621.

## Introduction

Osteoarthritis (OA) is characterized by progressive loss of joint cartilage that eventually leads to degradation of many important components of the joint. Damage from mechanical stress with insufficient self-repair by joints is believed to be the primary cause of OA (1). OA is the leading cause of chronic pain and disability worldwide (2). Owing to its negative impact on individual functioning and health service expenditure, OA has been designated a National Health Priority area in Australia (3). In 2012 it was estimated that 1.9 million people in Australia had OA, and this figure is predicted to increase to 3.0 million people by 2032 (4).

Knee OA is a very common subtype of OA with prevalence increasing with age. The global prevalence of symptomatic knee OA in 2010 was estimated to be 3.8%, with prevalence peaking at ∼50 y of age (5). Inflammation occurs locally within joints and is associated with knee pain severity in knee OA (6). Nonsurgical management of OA primarily involves the use of nonsteroidal anti-inflammatory drugs (NSAIDs) (7). However, owing to the adverse effects associated with long-term NSAID use, there is a need to identify alternative therapies that can safely and effectively reduce pain and inflammation and improve function in people with knee OA. Australian research has reported that omega-3 fatty acid supplements are commonly used for the management of OA, particularly among females with OA (8, 9), presumably owing to the known anti-inflammatory effects of ω-3 fatty acids and derivatives.

Krill, *Euphausia superba*, are small marine crustaceans, reported to be the largest biomass in the world, with an estimated 300,000 million metric tons located in the Antarctic Ocean (10). Krill are rich in the long-chain (LC) ω-3 PUFAs EPA (20:5n–3) and DHA (22:6n–3) and the antioxidant astaxanthin (11), which have known anti-inflammatory effects (12, 13). Structurally, krill oil differs from other dietary sources of LC ω-3 PUFAs in that it contains a relatively high amount of LC ω-3 PUFAs from phospholipids rather than triglycerides, which are the primary source of EPA and DHA found in fish oil (11). Some evidence suggests that the higher relative phospholipid content of krill oil may facilitate the incorporation of LC ω-3 PUFAs into tissues more efficiently than fish oil (11, 14). However, Yurko-Mauro et al. (15) showed no difference in plasma and RBC concentrations of EPA and DHA between fish oil and krill oil products when matched for dose, EPA, and DHA concentrations in a 4-wk randomized controlled trial (RCT). Preliminary data suggest that supplementation with krill oil is well tolerated in humans with only minor adverse effects reported, and may improve knee pain associated with OA (16, 17). However, given the methodological limitations of these trials, high-quality RCTs are warranted to investigate the efficacy of krill oil on knee pain associated with OA.

The primary objective of the study was to evaluate the efficacy of 4 g of a commercially available krill oil supplement daily on pain reduction in adults with mild to moderate OA of the knee compared with a placebo over a 6-mo period. The secondary outcomes included knee stiffness, knee physical function, serum lipid profiles (triglycerides, total cholesterol, LDL cholesterol, HDL cholesterol), Omega-3 Index, serum inflammatory markers [high-sensitivity C-reactive protein (hsCRP), IL-6, TNF-α], NSAID use, and safety markers.

## Methods

The trial was conducted at 4 sites across Australia. Human Research Ethics Committee approvals were obtained from the Commonwealth Scientific and Industrial Research Organisation (CSIRO) Human Research Ethics Committee (Adelaide, Australia) (reference number: 2/2017) and Bellberry Limited (Adelaide, Australia) (reference number: 2018-01-046). The trial was prospectively registered with clinicaltrials.gov (NCT03483090) and conducted in accordance with International Council on Harmonisation Good Clinical Practice (ICH-GCP) guidelines. The intervention phase of the study was executed from 27 February, 2018 to 30 December, 2019. The **Supplemental Methods** summarize the changes to the methods after trial commencement.

### Participants

Participants were recruited via social media and local advertisements. Oral and written information about the study objectives and protocol were provided to each individual and written informed consent was obtained before performing any study-related assessments.

Inclusion criteria: male or female; 40–65 y old inclusive; clinical diagnosis of OA of the index knee according to American College of Rheumatology (ACR) criteria for the classification of idiopathic OA of the knee; Kellgren-Lawrence (KL) grade 1–3 of the index knee, evidenced by knee X-ray; self-reported pain in the index knee on ≥4 d/wk for the last 3 mo; pain of the index knee between 4 and 8 cm (inclusive) over the 7 d before baseline as self-assessed on a 10-cm visual analog scale (VAS) (see the Supplemental Methods for more details on clinical diagnosis of OA of the knee and knee pain–related assessments); BMI (in kg/m^2^) >18.5 and <35; willingness to abstain from use of restricted medications; habitual intake of LC ω-3 PUFAs (from food and supplements) <500 mg/d as assessed using the validated Australian PUFA FFQ (18) and willingness to maintain a low intake throughout the study; and willing to provide written informed consent.

Exclusion criteria: severe radiographic knee OA in any knee defined as KL grade > 3; conditions which could interfere with the evaluation of the index knee; history of Reiter syndrome, rheumatoid arthritis, psoriatic arthritis, ankylosing spondylitis, arthritis associated with inflammatory bowel disease, sarcoidosis, amyloidosis, or any other forms of inflammatory arthritis; history of or clinical signs and symptoms of infection in the index joint; knee pain not clinically attributable to OA of the knee; pain in any other area of the lower extremities or back that was equal to or greater than the index knee pain (based on self-report); arthroscopy or open knee surgery in the index knee in the previous 12 mo or planned for within the duration of the study period; joint-related intraarticular (IA), intramuscular (IM), or oral interventions or therapies; bleeding disorders, taking anticoagulants; regular use of and not prepared to abstain from glucosamine, fish oil, curcumin, and other complementary medicines/supplements that may affect the study results; positive urine dipstick pregnancy test, currently pregnant and/or breastfeeding; females of child-bearing potential (FOCBP) not using effective methods of contraception; history of or known presence of alcohol abuse or illicit drug use (including cannabis); any surgical history, clinically significant conditions, organ dysfunction, recent or planned hospitalizations, or investigational drug consumption within 3 mo of baseline that may have affected the participant's ability to participate in the study or the study results; known or suspected allergies to the investigational products; history of an adverse reaction or known hypersensitivity to seafood or shellfish; and uncontrolled hypertension.

### Study design and procedures

The trial design was a 6-mo multicenter, randomized, placebo-controlled, double-blind, parallel-arm, phase II study. Eligible participants were randomly assigned to treatment groups in a 1:1 ratio using the method of minimization via an interactive voice response system (IVRS) [National Health and Medical Research Council (NHMRC) Clinical Trials Centre Central Randomisation Service]. Minimization variables were study site, gender, VAS knee pain score (4–5; 6–8), and age (40–49; 50–59; ≥60 y). Treatment allocation was concealed from study staff by having treatments sealed in identical opaque containers and numbered with sequential kit numbers according to the allocation sequence. All participants, study staff, and statisticians were blinded to treatment allocations until after the statistical analyses were completed.

**Supplemental Table 1** outlines the schedule of assessments. After telephone prescreening, participants attended a clinic screening visit to assess eligibility. On day 1 (baseline) participants returned to the clinic for confirmation of eligibility, informed consent, randomization, and baseline assessments. In-clinic study assessments were also completed on day 85 (3 mo) and day 169 (6 mo). After each of the baseline and 3-mo visits participants were discharged from the clinic with a compliance and medication checklist, a 3-mo supply of study treatment, and instructions. Online surveys were conducted at 1, 2, 4, and 5 mo to assess treatment compliance, adverse events (AEs), and use of concomitant medications. Any queries from the surveys were followed up by phone call. A final participant online survey and follow-up phone call (as needed) were conducted 28 d after the 6-mo study visit for a final safety assessment including a review of AEs and concomitant medications. In the event of early withdrawal from the study participants were encouraged to return to the clinic as soon as possible for an early withdrawal visit (similar to the 6-mo visit). Participants were requested, where possible, to maintain stable doses of concomitant medications. The following medications were prohibited during the study: anticoagulants and antiplatelet medications (except low-dose aspirin), high-dose NSAIDs, IM/IA corticosteroids to either knee, any IA intervention or therapy, regular oral corticosteroids, other investigational treatments, opioids, and opiates (see the Supplemental Methods for more detail).

Compliance to consumption of study treatments was defined as the number of capsules consumed over the 6-mo (169 d) period as a percentage of the number of capsules that should have been consumed over the 6-mo period.

Medical and surgical histories were obtained from the participant by a medical investigator through a physical examination and interview. Height was measured using a stadiometer (SECA) and body weight using calibrated electronic digital scales (Mercury, AMZ 14). BMI was calculated. A urine dipstick pregnancy test was performed in FOCBP at screening and all study visits.

### Investigational products

The commercial krill oil [“Swisse High Strength Deep Sea Krill Oil” (Superba^TM^ BOOST, Aker BioMarine)] contained, per capsule, 1 g krill oil (*E. superba* oil; 0.15 g EPA, 0.07 g DHA, of which 73% of EPA and DHA was bound to phospholipids, and 0.11 mg astaxanthin) in a black, oblong natural soft gelatin capsule. Each placebo capsule, matched to the krill oil in appearance and odor, contained 1 g mixed vegetable oil (olive oil, corn oil, palm oil, and medium-chain triglycerides) comprising 31% SFAs, 46% MUFAs, and 22% PUFAs, with no detectable EPA or DHA. The mixture of dietary fatty acids reflected the normal diet, and no single type of fatty acid or fat source was over-represented in the placebo that may have had independent therapeutic effects. Within the context of the whole diet in which fat provides ∼30% of total energy intake (%E), the small amount of mixed fats that were consumed as placebo (1.7%E) was unlikely to have any independent therapeutic effects.

Participants were randomly assigned to consume 4 capsules/d of either krill oil [providing in total 0.88 g/d EPA + DHA (0.60 g EPA, 0.28 g DHA) and 0.45 mg astaxanthin] or placebo. They were instructed to consume all 4 capsules at 1 occasion every day with or immediately after a meal and to return any unused study capsules and packaging at their next visit for determining compliance to study treatments.

The krill oil dosage was set higher than those used in previous studies on knee OA so as to facilitate an anti-inflammatory response, but in amounts suitable as a complementary medicine. EPA + DHA dosages typically proposed to exert anti-inflammatory responses have been >2 g/d, which fall into the pharmacologic range and are better achieved through pharmaceutical preparations (19) than complementary medicines. Given that most of the EPA and DHA in krill oil are delivered in phospholipid form, which may facilitate more efficient incorporation of LC ω-3 PUFAs into tissues than triglyceride form (11, 14), and that krill oil contains astaxanthin which confers additional anti-inflammatory effects (13), we proposed that krill oil's anti-inflammatory effect would be greater than what would be expected based on its LC ω-3 PUFA content alone.

### Knee pain, stiffness, and physical function

Knee pain (primary outcome), stiffness, and physical function (secondary outcomes) of the index knee were assessed using the validated Western Ontario and McMaster Universities Osteoarthritis Index (WOMAC) (20). The WOMAC Questionnaire was self-administered in paper-based format and comprised 24 items, each with a numeric scale response of 0 (no pain) to 10 (extreme pain).

Responses to items within each subscale were summed to create a WOMAC knee pain score (0–50), WOMAC knee stiffness score (0–20), WOMAC knee physical function score (0–170), and WOMAC global score (0–240). Raw scores were rescaled to a 0–100 scale (21). See the Supplemental Methods for more detail.

### Biochemical assessments

Serum was obtained from fasting venous blood samples collected in vacutainer tubes containing clot activator and left at room temperature for 30 min to allow for clot formation. The blood was then centrifuged (GS-6R centrifuge; Beckman Coulter Inc.) for 15 min at 2850 x g at 4°C. The resultant serum was divided into aliquots and stored at −70°C until analysis at the end of the intervention. Samples from each participant were analyzed within the same analytic run to reduce variation.

Serum lipid variables (total cholesterol, HDL cholesterol, and triglycerides) and hsCRP were analyzed on a Beckman AU480 clinical analyzer (Beckman Coulter Inc.) using commercial enzymatic test kits. LDL cholesterol was calculated using the Friedewald equation. Serum IL-6 and TNF-α were analyzed using the Luminex 100/200 system with xPONENT software (Luminex) and commercial assay kits. Intra-assay CVs were as follows: TC, 0.78%; triglycerides, 0.86%; HDL cholesterol, 0.69%; hsCRP, 2.36%; IL-6, 9.9%; TNF-α, 6.8%.

Omega-3 Index analysis was conducted by OmegaQuant (OmegaQuant LLC) as described by Harris and Polreis (22). In brief, Omega-3 Index phlebotomy kits (OmegaQuant LLC) approved by the Australian Therapeutic Goods Administration (ARTG 277814) were used to collect finger prick dried blood spot samples. The dried blood spot cards were stored at −70°C until fatty acid analysis at the end of the intervention. GC using a GC-2010 Gas Chromatograph (Shimadzu Corporation) equipped with a SP-2560, 100-m fused silica capillary column (0.25 mm internal diameter, 0.2 μm film thickness; Supelco) and an internal-standard-based 3-point calibration curve were used to quantify 24 fatty acids. The sum of the 24 fatty acids constituted the total fatty acid content of the blood and included SFAs [myristic acid (14:0), palmitic acid (16:0), stearic acid (18:0), arachidic acid (20:0), behenic acid (22:0), lignoceric acid (24:0)]; *cis* MUFAs [palmitoleic acid (16:1), oleic acid (18:1), gondoic acid (20:1), nervonic acid (24:1)]; *trans* unsaturated fatty acids [16:1, elaidic acid (18:1), linolelaidic acid (18:2)]; *cis* ω-6 PUFAs [linoleic acid (18:2), γ-linolenic acid (18:3), eicosadienoic acid (20:2), dihomo-γ-linolenic acid (20:3), arachidonic acid (20:4), adrenic acid (22:4), osbond acid (22:5)]; and *cis* ω-3 PUFAs [α-linolenic acid (18:3), EPA (20:5), docosapentaenoic acid (22:5), DHA (22:6)]. Each individual fatty acid was expressed as a percentage of the total fatty acids. The Omega-3 Index is defined as the sum of EPA and DHA content expressed as a percentage of total fatty acids in RBCs. Accordingly, the Omega-3 Index was calculated from the dried blood spot EPA + DHA value adjusted by a regression equation to predict the Omega-3 Index in RBCs. The laboratory CV for the dried blood spot Omega-3 Index is <5% (22).

### NSAID use

It was originally planned to calculate an NSAID equivalence score (23) using NSAID dosage and frequency data collected as part of reporting prior and concomitant medications. However, this was not feasible from the reported data because a large proportion of reported NSAIDs were taken pro re nata (PRN) (Latin for “as needed”), hence mean daily intakes could not be calculated for the majority of NSAID usage. Instead, post hoc exploratory analyses were undertaken, calculating the fraction of time over the 6-mo period where ≥1 NSAIDs were reportedly being used, either by PRN or by prescribed dosages and frequencies. For each participant who completed the 6-mo visit, any NSAID use (yes/no) was determined for each day of the 6-mo study period.

NSAID fraction was calculated as:

(1) $$\begin{equation*}
\frac{{\left[ {{\rm{total\ number\ of\ days\ where\ }} \ge 1{\rm{\ NSAIDs\ were\ reportedly\ used}}} \right]}}{{\left[ {{\rm{total\ number\ of\ study\ days\ between\ baseline\ and\ }}6 - {\rm{mo\ visit}}} \right]}}\nonumber\\
\end{equation*}$$

### Assessments of safety parameters

Resting blood pressure and pulse rate were measured using an automated blood pressure monitor with participants in a seated position after a 5-min rest. The mean of 3 measurements (separated by 2 min) was recorded. Respiratory rate was measured by counting the number of times the chest rose per minute while the participant was at rest. Body temperature was measured using a digital tympanic thermometer.

A noninvasive physical examination was performed by a medical investigator and included the following outcomes: general appearance; eyes, ears, nose, mouth, and throat; cardiovascular; respiratory; gastrointestinal/abdominal; musculoskeletal; skin. A symptom-directed physical examination was conducted at the 6-mo and early withdrawal visits.

Blood hematology, biochemistry, and coagulation outcomes were analyzed at screening, baseline, 3-mo, 6-mo, or early withdrawal visits by National Association of Testing Authorities, Australia (NATA) laboratories at each study site (see the Supplemental Methods for more detail).

#### AEs

Incidence of AEs and serious adverse events (SAEs) were recorded from baseline until the final safety visit or early withdrawal. At each clinic visit and in each online survey participants were questioned in a nonleading manner regarding the occurrence of any AE. For each AE its description, date of onset, duration, actions taken, outcome, and a medical investigator's opinion on severity and causality to study treatment were recorded. Use of all concomitant medications and abnormal laboratory values considered clinically significant by a medical investigator were recorded as an AE. All reported AEs/SAEs were coded using the latest version of the Medical Dictionary for Regulatory Activities (MedDRA; https://www.meddra.org). See the Supplemental Methods for more details on the methodology for assessing AEs.

### Data management and monitoring

Data collected during this study were handled, processed, and managed as per an approved study-specific data management plan and study monitoring was performed in accordance with applicable regulations, guidelines, and sponsor procedures. See the Supplemental Methods for more details on data management and monitoring.

### Statistical analysis

It was calculated that 238 participants would be required (1:1, 119 in each treatment group), allowing for a ∼20% dropout rate, to provide 80% power at a significance level of 5% to detect a standardized treatment effect of 0.4 (i.e., a medium effect) in WOMAC knee pain (24).

Statistical analyses were performed according to a detailed statistical analysis plan (SAP). Statistical analyses were performed using Stata®/SE software version 15.1 (StataCorp). Blinding of treatment allocations was maintained throughout the statistical analysis. No interim analysis was conducted. The primary outcome and all secondary outcomes were analyzed according to the intention-to-treat (ITT) principle, whereby all randomly assigned participants were included and analyzed according to the group to which they were originally assigned. As recommended by Jakobsen et al. (25) and Bennett (26), a complete case analysis (based on participants with a complete set of outcome data) was undertaken because the percentage of missing data in outcome variables was considered small (∼10%) and baseline covariate data were mostly complete. Per-protocol analyses were also conducted for selected outcomes, whereby only those randomly assigned participants who were compliant with the study protocol were included. Compliance was defined as consumption of ≥80% of the prescribed treatment on average over the 6-mo study period, and the absence of any major deviations from or violations of the protocol (e.g., use of prohibited medications). Analyses of safety outcomes were based on all randomly assigned individuals.

The level of statistical significance was set at 0.05 (2-sided). No multiple test adjustments were made for the number of secondary analyses performed because these analyses were considered exploratory.

#### Efficacy analysis

For continuously measured outcomes, distributional assumptions underpinning the planned statistical analysis were investigated and, if found to be violated, variables were log-transformed. All efficacy analyses were adjusted for stratification variables (site, gender, knee pain score, age at randomization). In addition, analyses of treatment efficacy were adjusted for baseline assessment of the specific outcome variable and the following 3 baseline factors believed to potentially affect the primary outcome (knee pain): BMI, OA severity, and the Omega-3 Index. Although VAS knee pain score was used as a stratification variable, baseline WOMAC knee pain score was used in the model instead to minimize potential collinearity.

For continuous variables, descriptive summaries included means ± SDs for normally distributed variables and medians [IQRs] for nonnormally distributed variables. Categoric variables were summarized using frequencies and percentages. Estimates of treatment effects are presented as adjusted mean change (95% CI) from baseline and differences in adjusted mean change (95% CI) from baseline between groups.

The primary analysis comparing change in WOMAC knee pain score from baseline at 6 mo between randomized groups was performed using ANCOVA, adjusted for prespecified covariates and using an ITT approach. Secondary analyses of the primary outcome extended the primary analysis to include *1*) an interaction term for treatment × baseline Omega-3 Index to assess evidence of effect modification by Omega-3 Index; *2*) inclusion of change in BMI from baseline as a covariate; and *3*) restricting the analysis to only those participants who were compliant with the protocol (per-protocol cohort). All secondary outcomes assessed at 2 time points (3 and 6 mo) were analyzed using linear mixed-effect models for each of the ITT and per-protocol analysis cohorts. Fixed effects were specified for treatment, assessment time (3 compared with 6 mo), the interaction of treatment × time, and the adjustment factors described already. An independence covariance matrix structure was assumed, and a random intercept for each participant included.

#### SAP deviations and efficacy-related post hoc analyses

Because baseline VAS knee pain score was replaced with baseline WOMAC knee pain score as an adjustment factor for the primary analyses of all efficacy outcomes, sensitivity analyses were conducted post hoc with the inclusion of VAS knee pain score as an additional covariate. Because the overall results did not change with inclusion of this covariate, the results of these analyses are not reported.

A large proportion of serum IL-6 values were recorded as 0 such that the prespecified linear mixed-effects model analysis was not performed. An exploratory analysis was conducted for each of the ITT and per-protocol analysis cohorts using random-effects tobit regression (censored regression) for log-transformed IL-6 values [all 0 values were set to 0.01, half the lowest nonzero value of IL-6 (0.02), before log transformation]. Fixed effects included treatment, time, the interaction of treatment × time, and the adjustment covariates (as aforementioned).

Because NSAID usage was mostly PRN, prespecified linear mixed-effects models to assess level of NSAID use could not be performed. Instead, a fractional regression model with a probit link was used to compare the mean fraction of study period whereby NSAIDs were used between treatment groups (27, 28). Adjustment variables included NSAID use at baseline (any compared with none) and other covariates as detailed already.

Because the main mechanism by which krill oil is hypothesized to affect OA pain is through anti-inflammatory effects, additional exploratory analyses were undertaken to determine whether effects of treatment on WOMAC knee outcomes were moderated by inflammatory status at baseline. Participants were categorized according to their baseline inflammatory status as follows: low (serum hsCRP <1 mg/L); medium (serum hsCRP ≥1 mg/L and ≤3 mg/L); and high (serum hsCRP >3 mg/L) inflammatory status (29). The primary analysis comparing change in WOMAC knee pain score from baseline to 6 mo between randomized groups using ANCOVA and an ITT approach was extended to include a treatment × baseline inflammatory status interaction term. Treatment effect modification by baseline inflammatory status was assessed for other WOMAC knee outcomes using a similar approach. Adjustment factors were as specified for the primary analysis. The overall significance of the treatment × baseline inflammatory status interaction was assessed using a Wald test. Estimates of treatment effect were obtained stratified by inflammatory group. Sensitivity analyses with hsCRP entered as a continuous variable were also performed.

For outcomes where linear mixed modeling was performed on log-transformed data, adjusted means and 95% CIs for the outcome in the log scale were back-transformed and presented as mean percentage changes with corresponding 95% CIs (see the Supplemental Methods for how estimates for mean percentage change were derived from estimates in the ln scale).

#### Safety analysis

For outcome variables that assessed presence or absence of a condition (e.g., AEs, SAEs, and physical examination), differences between treatment groups were analyzed using exact binomial tests and log binomial generalized linear regressions.

Differences between treatment groups for continuously measured outcome variables [vital signs (blood pressure, pulse rate, respiratory rate, temperature); hematology, biochemical, and coagulation parameters] were analyzed using linear mixed-effects models with fixed effects for treatment, time, and the interaction of treatment × time, adjusted for covariates as detailed already, and analysis adhered to the principles of ITT as closely as possible.

#### Deviations and post hoc analyses related to safety analysis

The prespecified linear mixed-effects model analysis was not performed for serum C-reactive protein (CRP) (distinct from serum hsCRP) because the data included a large proportion of censored observations. Serum CRP was instead redefined as a binary outcome with categories of <5 mg/L and ≥5 mg/L and analyzed using a generalized estimating equation log binomial regression model comparing the RR of CRP ≥5 mg/L between groups. Fixed effects included treatment, time, the interaction of treatment × time, and adjustment factors (as aforementioned) and analysis adhered to the ITT approach as closely as possible.

## Results

### Study population

**FIGURE 1:**
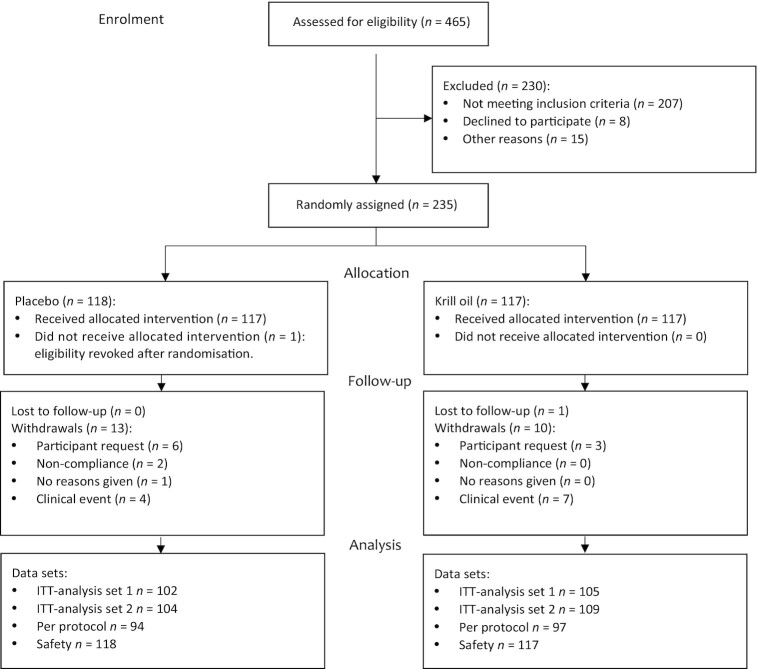
Flowchart of participants through the trial. ITT analysis set 1 for the primary analysis (ANCOVA) included all randomly assigned participants with a complete set of primary outcome (WOMAC knee pain at 6 mo) and covariate data. ITT analysis set 2 for the secondary analyses (linear mixed-effects models) included all randomly assigned participants with data available for ≥1 postrandomization time point and complete covariate data. Per-protocol analysis set included all participants in the ITT analysis set who were compliant with the study protocol. Safety data set included all randomly assigned participants. ITT, intention-to-treat; WOMAC, Western Ontario and McMaster Universities Osteoarthritis Index.

A total of 465 participants were screened (**Figure 1**). Of these, 235 participants were randomly assigned to treatment groups (krill oil: *n* = 117; placebo: *n* = 118), of whom 234 received allocated interventions (krill oil: *n* = 117; placebo: *n* = 117) and 24 (10%) withdrew early or were lost to follow-up (krill oil: *n* = 11; placebo: *n* = 13). No participant withdrew owing to knee replacement surgery.

The baseline characteristics summarized in **Table 1** reflect healthy males and females, aged 40–65 y with moderate OA and low Omega-3 Index levels. The treatment groups appeared balanced with respect to the reported characteristics. The majority of participants had a KL score of 3. Most participants did not use NSAIDs and usage was mostly reported to be PRN. Participants’ BMI remained relatively stable over the 6-mo study period (**Supplemental Table 2**).

**TABLE 1:** Baseline characteristics^1^

	Placebo (*n* = 118)	Krill oil (*n* = 117)	All (*n* = 235)
Male	54 (45.8)	52 (44.4)	106 (45.1)
Age, y	56.0 ± 6.8	55.8 ± 6.8	55.9 ± 6.8
Height, cm	172 ± 10.0	172 ± 9.9	172 ± 9.9
Body weight, kg	83.7 ± 14.1	83.7 ± 14.3	83.7 ± 14.2
BMI, kg/m^2^	28.4 ± 3.6	28.3 ± 3.8	28.3 ± 3.7
Systolic blood pressure, mm Hg	124 ± 13.4	121 ± 11.2	123 ± 12.4
Diastolic blood pressure, mm Hg	78.1 ± 7.9	77.8 ± 7.3	77.9 ± 7.6
Knee pain score (VAS)	5.3 ± 1.1	5.3 ± 1.1	5.3 ± 1.1
OA severity (Kellgren-Lawrence grade)			
1	26 (22.0)	22 (18.8)	48 (20.4)
2	36 (30.5)	32 (27.4)	68 (28.9)
3	56 (47.5)	63 (53.8)	119 (50.6)
Use of NSAID			
None	74 (62.7)	70 (59.8)	144 (61.3)
PRN only	28 (23.7)	35 (29.9)	63 (26.8)
Prescribed regular dose only	11 (9.3)	7 (6.0)	18 (7.7)
Prescribed regular dose + PRN	3 (2.5)	2 (1.7)	5 (2.1)
Missing	2 (1.7)	3 (2.6)	5 (2.1)
Omega-3 Index, %	5.5 ± 0.9	6.0 ± 1.3	5.8 ± 1.2
Inflammatory status^2^			
Low (<1 mg/L hsCRP)	38 (32.2)	46 (39.3)	84 (35.7)
Medium (≥1 mg/L to ≤3 mg/L hsCRP)	49 (41.5)	48 (41.0)	97 (41.3)
High (>3 mg/L hsCRP)	29 (24.6)	22 (18.8)	51 (21.7)

1 All values are mean ± SD or *n* (%) unless otherwise indicated. hsCRP, high-sensitivity C-reactive protein; NSAID, nonsteroidal anti-inflammatory drug; OA, osteoarthritis; PRN, pro re nata (Latin for “as needed”); VAS, visual analog scale.

2 Classification system described by the CDC and the American Heart Association (29).

### Compliance

Participant compliance was excellent with >80% of participants consuming ≥80% of the study treatment on average over the 6-mo study period, and 80% of participants compliant overall (**Supplemental Table 3**).

A total of 405 protocol deviations were reported, with the majority being minor and unlikely to have affected either the safety of participants or the study outcomes.

### Omega-3 Index

The mean Omega-3 Index increased in the krill oil group from 6.0% at baseline to 8.9% at 3 mo and 9.0% at 6 mo, whereas mean levels remained stable, between 5.4% and 5.5%, in the placebo group over time (**Supplemental Table 4**). Omega-3 Index was estimated to have increased by 3.11% (95% CI: 2.86%, 3.37%) more in the krill oil group than in the placebo group at 3 mo, and by 3.22% (95% CI: 2.96%, 3.48%) more in the krill oil group than in the placebo group at 6 mo, after controlling for prespecified covariates (**Table 2**). Similar results were observed in the per-protocol analysis set.

**TABLE 2:** Adjusted mean (95% CI) changes in Omega-3 Index (%) from baseline and comparisons between treatment groups^1^

	Intention-to-treat				Per protocol			
	Placebo (*n* = 104)	Krill oil (*n* = 109)	Krill oil vs. placebo	*P* value^2^	Placebo (*n* = 94)	Krill oil (*n* = 96)	Krill oil vs. placebo	*P* value^2^
Δ 3 mo	−0.15 (−0.33, 0.03)	2.97 (2.79, 3.14)	3.11 (2.86, 3.37)	<0.001	−0.17 (−0.37, 0.02)	3.01 (2.82, 3.20)	3.18 (2.90, 3.46)	<0.001
Δ 6 mo	−0.18 (−0.37, 0.00)	3.04 (2.86, 3.22)	3.22 (2.96, 3.48)	<0.001	−0.22 (−0.41, −0.02)	3.08 (2.89, 3.27)	3.30 (3.02, 3.58)	<0.001

1 Δ, change from baseline.

2 Comparisons between treatment groups were performed using linear mixed-effects models on intention-to-treat (*n* = 221*) and per-protocol data sets (*n* = 190); changes from baseline were calculated by subtracting 3- and 6-mo data from baseline data and compared while controlling for baseline Omega-3 Index, study site, gender, baseline Western Ontario and McMaster Universities Osteoarthritis Index knee pain score, age, BMI, and osteoarthritis severity. *The analysis population included all randomly assigned participants with data available for ≥1 postrandomization time point.

### Knee pain, stiffness, and physical function

**Supplemental Table 5** summarizes descriptive statistics for WOMAC knee outcomes at baseline, 3 mo, and 6 mo, and for change from baseline at 3 mo and 6 mo.

**FIGURE 2:**
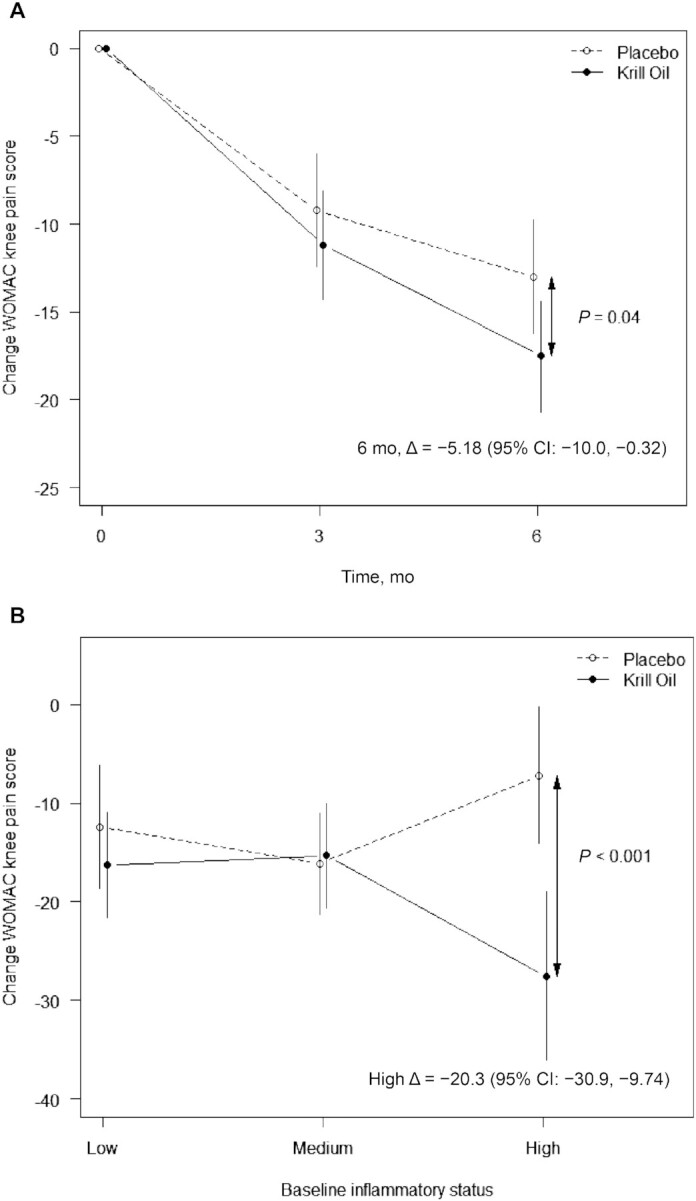
Adjusted mean (95% CI) changes in WOMAC knee pain scores from baseline at 6 mo. (A) Primary analysis comparing changes in WOMAC knee pain score (ITT population, *n* = 207, *P* = 0.04). (B) Secondary analysis comparing changes in WOMAC knee pain scores stratified by baseline inflammatory status (ITT approach, *n* = 205, overall treatment × inflammatory status interaction *P* = 0.01). WOMAC knee pain scores were normalized to scores ranging from 0 to 100. Changes from baseline were calculated by subtracting 3-mo and 6-mo data from baseline data and compared using ANCOVA while controlling for baseline WOMAC knee pain score, study site, gender, Omega-3 Index, age, BMI, and osteoarthritis severity. The analysis population included all randomly assigned participants with a 6-mo assessment and complete covariate data. ITT, intention-to-treat; WOMAC, Western Ontario and McMaster Universities Osteoarthritis Index; Δ, change from baseline.

The primary ITT analysis of the change in WOMAC knee pain score from baseline at 6 mo (primary outcome) using ANCOVA showed that WOMAC knee pain had greater decreases in the krill oil group (estimated change: −17.8; 95% CI: −21.2, −14.4) than in the placebo group (estimated change: −12.6; 95% CI: −16.0, −9.2) with the difference between groups being −5.18 (95% CI: −10.0, −0.32) in favor of the krill oil group (*P* = 0.04) (**Figure 2**A). Inclusion of either an interaction term for treatment × baseline Omega-3 Index or a covariate for change in BMI from baseline did not substantially alter the direction, magnitude, or significance of the estimated treatment effect for the primary outcome. The estimated treatment effects at 6 mo from secondary analyses using linear mixed models were of similar magnitude, for both the ITT and per-protocol analysis sets (**Table 3**). Differences between groups at 3 mo were not statistically significant (Table 3).

**TABLE 3:** Adjusted mean (95% CI) changes in WOMAC knee outcomes from baseline and comparisons between treatment groups^1^

	Intention-to-treat				Per protocol			
	Placebo (*n* = 104)	Krill oil (*n* = 109)	Krill oil vs. placebo	*P* value^2^	Placebo (*n* = 94)	Krill oil (*n* = 96)	Krill oil vs. placebo	*P* value^2^
WOMAC knee pain score								
Δ 3 mo	−9.20 (−12.4, −6.02)	−11.2 (−14.3, −8.10)	−2.00 (−6.50, 2.50)	0.38	−9.00 (−12.3, −5.72)	−11.5 (−14.7, −8.24)	−2.46 (−7.14, 2.20)	0.30
Δ 6 mo	−13.0 (−16.2, −9.76)	−17.5 (−20.7, −14.4)	−4.60 (−9.12, −0.06)	0.047	−13.0 (−16.3, −9.76)	−18.2 (−21.4, −15.0)	−5.18 (−9.84, −0.50)	0.03
WOMAC knee stiffness score								
Δ 3 mo	−13.7 (−17.6, −9.80)	−13.2 (−17.0, −9.40)	0.45 (−5.00, 5.95)	0.87	−13.8 (−17.9, −9.70)	−13.7 (−17.7, −9.60)	0.15 (−5.65, 6.00)	0.95
Δ 6 mo	−13.1 (−17.0, −9.10)	−19.5 (−23.4, −15.7)	−6.45 (−12.1, −0.9)	0.02	−13.2 (−17.3, −9.15)	−20.1 (−24.6, −16.5)	−7.35 (−13.2, −1.55)	0.01
WOMAC knee physical function score								
Δ 3 mo	−7.85 (−11.1, −4.60)	−10.0 (−13.2, −6.90)	−2.21 (−6.79, 2.37)	0.34	−7.20 (−10.6, −3.81)	−10.4 (−13.8, −7.08)	−3.23 (−8.08, 1.61)	0.19
Δ 6 mo	−10.1 (−13.4, −6.90)	−14.8 (−18.1, −11.6)	−4.67 (−9.26, −0.05)	0.047	−10.1 (−13.6, −6.79)	−15.9 (−19.3, −12.6)	−5.76 (−10.6, −0.92)	0.02
WOMAC knee total score								
Δ 3 mo	−8.65 (−11.8, −5.50)	−10.6 (−13.7, −7.52)	−1.95 (−6.43, 2.55)	0.39	−8.19 (−11.5, −4.83)	−11.0 (−14.3, −7.73)	−2.83 (−7.56, 1.92)	0.24
Δ 6 mo	−11.0 (−14.3, −7.85)	−15.9 (−19.0, −12.7)	−4.83 (−9.37, −0.27)	0.04	−11.1 (−14.4, −7.73)	−16.9 (−20.2, −13.6)	−5.84 (−10.6, −1.09)	0.02

1 WOMAC scores were normalized to scores ranging from 0 to 100. WOMAC, Western Ontario and McMaster Universities Osteoarthritis Index; Δ, change from baseline.

2 Comparisons between treatment groups were performed using linear mixed-effects models on intention-to-treat (*n* = 213)* and per-protocol populations (*n* = 190); changes from baseline were calculated by subtracting 3- and 6-mo data from baseline data, respectively, and compared while controlling for the baseline level of the respective outcome variable, study site, gender, baseline WOMAC knee pain score, Omega-3 Index, age, BMI, and osteoarthritis severity. *The analysis population included all randomly assigned participants with data available for ≥1 postrandomization time point.

Other WOMAC outcomes, including knee stiffness, physical function, and WOMAC knee total scores, also showed significantly greater improvements after 6 mo with krill oil than with placebo in both ITT and per-protocol analyses (Table 3).

There was some evidence for treatment effect modification by baseline inflammatory status with respect to change in WOMAC knee pain score from baseline at 6 mo (Figure 2B, **Supplemental Table 6**). The estimated treatment effect was greater in the high inflammatory group (serum hsCRP >3 mg/L) than in both the low (hsCRP <1 mg/L) and medium (hsCRP ≥1 mg/L and ≤3 mg/L) inflammatory groups: −20.3 (95% CI: −30.9, −9.74) compared with −3.88 (95% CI: −12.0, 4.24) and 0.82 (95% CI: −6.56, 8.20), respectively. When baseline hsCRP was entered into the model as a continuous variable (sensitivity analysis), the interaction with treatment on change in WOMAC knee pain score at 6 mo remained significant (*P* = 0.03) (**Supplemental Table 7**). The estimated treatment effect in the high inflammatory group was not as pronounced as when hsCRP risk levels were entered as a categorical variable (−11.8; 95% CI: −19.7, −3.98), which may be because the latter model assumes a linear relation between baseline hsCRP and change in pain score, whereas the effect of baseline hsCRP appears to be nonlinear. Treatment effect modification by baseline inflammatory status was also assessed for the other WOMAC outcomes of stiffness, physical function, and WOMAC total score, but the treatment × inflammatory status interaction was not significant for these outcomes. Trends toward greater treatment effects in the high inflammatory group than in the low and medium inflammatory groups were observed for change in WOMAC knee physical function and WOMAC total score (Supplemental Table 6).

### NSAID use

NSAIDs (oral or topical; below the prohibited medication limits) were estimated as having been used on 39% and 38% of the study days over the 6-mo study period, mostly PRN, by participants in the placebo and krill oil groups, respectively [adjusted mean fractions of study days where NSAIDs were used: 0.39; 95% CI: 0.38, 0.40 and 0.38; 95% CI: 0.37, 0.39 in the placebo (*n* = 102) and krill oil (*n* = 105) groups, respectively; effect estimate for krill oil compared with placebo: −0.01; 95% CI: −0.03, 0.01; *P* = 0.24].

### Serum lipids

**Supplemental Table 8** summarizes descriptive statistics for serum lipid outcomes at baseline, 3 mo, and 6 mo, and for change from baseline at 3 mo and 6 mo.

Changes in serum total cholesterol, HDL cholesterol, and triglycerides from baseline did not significantly differ between treatment groups in either of the ITT or per-protocol analyses (**Table 4**). A small (5%) increase in LDL cholesterol from baseline at 3 mo in the krill oil group compared with the placebo group was detected in both ITT and per-protocol analyses. The effect was transient because at 6 mo the groups did not differ (Table 4).

**TABLE 4:** Adjusted mean (95% CI) changes in serum lipid outcomes from baseline and comparisons between treatment groups^1^

	Intention-to-treat				Per protocol			
	Placebo (*n* = 109)	Krill oil (*n* = 112)	Krill oil vs. placebo	*P* value^2^	Placebo (*n* = 93)	Krill oil (*n* = 95)	Krill oil vs. placebo	*P* value^2^
Total cholesterol, mmol/L								
Δ 3 mo	0.00 (−0.12, 0.13)	0.17 (0.04, 0.29)	0.16 (−0.01, 0.34)	0.07	0.01 (−0.13, 0.15)	0.19 (0.05, 0.32)	0.18 (−0.02, 0.37)	0.08
Δ 6 mo	−0.02 (−0.15, 0.11)	0.03 (−0.09, 0.16)	0.05 (−0.13, 0.23)	0.56	0.00 (−0.14, 0.14)	0.08 (−0.05, 0.22)	0.08 (−0.12, 0.28)	0.43
HDL-C, mmol/L								
Δ 3 mo	0.02 (−0.01, 0.05)	0.06 (0.03, 0.09)	0.04 (−0.01, 0.08)	0.12	0.02 (−0.02, 0.05)	0.05 (0.02, 0.09)	0.04 (−0.01, 0.08)	0.13
Δ 6 mo	0.02 (−0.01, 0.05)	0.05 (0.02, 0.08)	0.03 (−0.02, 0.07)	0.21	0.03 (−0.01, 0.06)	0.05 (0.02, 0.08)	0.02 (−0.02, 0.07)	0.32
LDL-C, mmol/L								
Δ 3 mo	−0.03 (−0.14, 0.08)	0.17 (0.06, 0.28)	0.20 (0.05, 0.36)	0.01	−0.03 (−0.15, 0.10)	0.19 (0.07, 0.31)	0.22 (0.04, 0.39)	0.01
Δ 6 mo	−0.05 (−0.16, 0.06)	0.02 (−0.09, 0.13)	0.07 (−0.09, 0.23)	0.38	−0.02 (−0.14, 0.10)	0.06 (−0.06, 0.18)	0.08 (−0.09, 0.26)	0.34
Triglycerides,^3^ %								
Δ 3 mo	0.67 (−5.16, 6.87)	−6.45 (−11.82, −0.75)	−7.07 (−14.63, 1.15)	0.09	1.08 (−5.31, 7.89)	−6.00 (−11.90, 0.30)	−7.00 (−15.26, 2.07)	0.13
Δ 6 mo	0.60 (−5.31, 6.89)	−3.26 (−8.84, 2.66)	−3.84 (−11.73, 4.76)	0.37	0.01 (−6.31, 6.75)	−3.03 (−9.09, 3.43)	−3.04 (−11.63, 6.39)	0.51

1 HDL-C, high-density lipoprotein cholesterol; LDL-C, low-density lipoprotein cholesterol; Δ, change from baseline.

2 Comparisons between treatment groups were performed using linear mixed-effects models on intention-to-treat (*n* = 221)* and per-protocol data sets (*n* = 188); changes from baseline were calculated by subtracting 3- and 6-mo data from baseline data and compared while controlling for the baseline level of the respective outcome variable, study site, gender, baseline Western Ontario and McMaster Universities Osteoarthritis Index knee pain score, Omega-3 Index, age, BMI, and osteoarthritis severity. *The analysis population included all randomly assigned participants with data available for ≥1 postrandomization time point.

3 Statistical analyses were performed on log-transformed data; adjusted means and 95% CIs on the log scale were back-transformed and are presented as mean percentage change values.

### Serum inflammatory outcomes

**Supplemental Table 9** summarizes descriptive statistics for serum inflammatory outcomes at baseline, 3 mo, and 6 mo, and for change from baseline at 3 mo and at 6 mo.

No significant differences in changes in serum inflammatory markers from baseline were detected between treatment groups, in either of the ITT or per-protocol analyses (**Table 5**).

**TABLE 5:** Adjusted mean (95% CI) percentage changes in serum inflammatory outcomes from baseline and comparisons between treatment groups^1^

	Intention-to-treat				Per protocol			
	Placebo (*n* = 109)	Krill oil (*n* = 112)	Krill oil vs. placebo	*P* value^2^	Placebo (*n* = 93)	Krill oil (*n* = 95)	Krill oil vs. placebo	*P* value^2^
IL-6,^3^ %								
Δ 3 mo	189 (−4.93, 777)	46.8 (−50.4, 335)	−49.2 (−89.1, 138)	0.39	280 (8.01, 1234)	57.7 (−52.9, 428)	−58.5 (−92.6, 135)	0.32
Δ 6 mo	169 (−13.4, 737)	195 (0.02, 770)	9.56 (−76.9, 419)	0.90	172 (−23.3, 862)	250 (6.75, 1047)	28.9 (−77.0, 621)	0.77
TNF-α, %								
Δ 3 mo	−5.42 (−19.4, 11.0)	3.09 (−12.0, 20.8)	9.00 (−13.2, 36.8)	0.46	−0.51 (−15.7, 17.4)	3.51 (−12.2, 22.0)	4.04 (−17.9, 31.8)	0.74
Δ 6 mo	−2.22 (−16.9, 15.0)	5.37 (−10.2, 23.6)	7.76 (−14.4, 35.6)	0.52	6.12 (−10.1, 25.2)	7.85 (−8.44, 27.0)	1.63 (−19.7, 28.6)	0.89
High-sensitivity C-reactive protein, %								
Δ 3 mo	−5.00 (−15.2, 6.40)	−1.95 (−12.3, 9.67)	3.21 (−12.1, 21.2)	0.70	−4.82 (−15.6, 7.39)	−1.74 (−12.8, 10.8)	3.23 (−13.1, 22.6)	0.72
Δ 6 mo	−3.26 (−13.8, 8.50)	−0.29 (−10.9, 11.6)	3.07 (−12.4, 21.2)	0.72	−3.37 (−14.4, 9.02)	0.10 (−11.2, 12.8)	3.58 (−12.8, 23.0)	0.69

1 All statistical analyses were performed on log-transformed data; adjusted mean (95% CI) log data were back-transformed and are presented as mean (95% CI) percentage values. Δ, change from baseline.

2 Comparisons between treatment groups were performed using linear mixed-effects models on intention-to-treat (*n* = 229)* and per-protocol data sets (*n* = 188); changes from baseline were calculated by subtracting 3- and 6-mo data from baseline and compared while controlling for the baseline level of the respective outcome variable, study site, gender, baseline Western Ontario and McMaster Universities Osteoarthritis Index knee pain score, Omega-3 Index, age, BMI, and osteoarthritis severity. *The analysis population included all randomly assigned participants with data available for ≥1 postrandomization time point.

3 Because a large proportion of IL-6 values were recorded as 0, random-effects tobit regression (censored regression) was performed [all 0 values were set to a value of 0.01, which was equal to half the lowest observed value of IL-6 (0.02), before log transformation]. Covariates were as described in the text.

Treatment effect modification by baseline inflammatory status (hsCRP-based categories) was assessed with respect to change in hsCRP from baseline. The interaction was not significant (*P* = 0.07) (**Supplemental Table 10)**, so there is insufficient evidence that the effect of treatment on the change in hsCRP from baseline differed depending on baseline hsCRP.

### Safety assessments

**Supplemental Table 11** summarizes descriptive statistics for vital sign outcomes at baseline, 3 mo, and 6 mo. Vital sign outcomes remained stable over time in both treatment groups and there were no significant differences between groups at any time point (**Supplemental Table 12**).

The number of unsatisfactory assessments of physical examination outcomes was generally low. Observed differences between groups for musculoskeletal and kidney and bladder outcomes were not statistically significant, indicating these differences were not beyond what would be expected by chance (**Supplemental Table 13**).

**Supplemental Table 14** summarizes descriptive statistics for serum biochemistry, hematology, and coagulation outcomes at baseline, 3 mo, and 6 mo. The mean (or median) levels of these variables were all within normal reference ranges throughout the study period. Most of these outcomes did not differ between treatment groups (**Supplemental Table 15**). Statistically significant (*P* < 0.05) differences between groups were detected for a small number of outcomes (7 out of 34 variables). The differences between groups in change from baseline were generally modest (<10% from baseline) and unlikely to be of clinical relevance. Bicarbonate decreased slightly with placebo compared with krill oil at 6 mo. A small increase was seen in glucose after 3 (∼4%) and 6 mo (∼5%) with krill oil compared with placebo. Uric acid decreased slightly (∼2%) with krill oil, whereas it increased slightly with placebo (∼3%) at 6 mo. Similarly, phosphate increased slightly with krill oil (∼2%) and decreased slightly with placebo (∼2%). Albumin was reduced in both krill oil and placebo groups, but the decrease at 6 mo was slightly greater with krill oil than with placebo (∼2% and ∼1%, respectively). Globulin decreased after 3 mo with placebo (∼2%), but at 6 mo the groups did not differ. At 6 mo alanine aminotransferase concentrations increased with krill oil (5.7%) and decreased with placebo (3%). Any outcomes deemed clinically important by the principal investigator/medical investigator that arose from the biochemistry, hematology, or coagulation reports were also captured as AEs.

A total of 155 AEs and 4 SAEs were reported, with incidence approximately equal across the 2 treatment groups (**Supplemental Table 16**). The most common AEs were upper respiratory tract infections, nasopharyngitis (cold), joint (arthralgia) and back pain, gastrointestinal disorders (e.g., diarrhea), and headaches (**Supplemental Table 17**). Incidence of treatment-related AEs was low and did not differ between treatment groups (**Supplemental Table 18**). None of the SAEs were reported to be treatment related (Supplemental Tables 16 and **19**). No pregnancies were reported.

## Discussion

This RCT represents the largest, longest, and highest-dose study to date investigating the effects of krill oil on OA knee pain. The main results showed a significant increase in Omega-3 Index to 9% after 6 mo of treatment, accompanied by a modest reduction in knee pain as well as knee stiffness and improvement in physical function as compared with placebo.

Previous studies have reported improved OA symptoms with krill oil consumption (16, 17). However, these studies were generally small, of short duration (30 d), and used very low dosages. Suzuki et al. (17) showed in a population with mild knee pain small improvements with krill oil [2 g/d (0.35 g/d EPA + DHA)] compared with placebo in only 3 out of ∼30 individual scores related to joint pain using the Japanese Knee OA Measure and Japanese Orthopedic Association score. Deutsch (16) recruited participants with diagnosed cardiovascular disease and/or rheumatoid arthritis and/or OA with increased CRP concentrations. Both CRP concentrations and WOMAC pain scores decreased after 7 d and continued to decrease over the 30-d trial compared with placebo. Hill et al. (24) showed, in their 2-y RCT of 202 participants with knee OA and regular knee pain, that a low dosage of EPA + DHA (0.3 g/d) was more effective in improving WOMAC pain and function scores than a high dosage (4.5 g/d EPA + DHA) (24). The authors were unable to explain this finding and no placebo was provided against which the low-dose fish oil could be compared.

Krill oil is suggested to reduce pain through anti-inflammation mechanisms, due to its high content of LC ω-3 PUFAs (12) and astaxanthin (13). EPA and DHA influence inflammation through various mechanisms, including modulation of the proinflammatory eicosanoids toward a more anti-inflammatory profile (30); and through the generation of proresolving lipid mediator compounds including resolvins, protectins, and maresins (12). In particular, the D- and E-series resolvins, derived from DHA and EPA, respectively, have been demonstrated to play roles in attenuating inflammatory-related pain (31, 32). In animal models of OA, activation of the D-series resolving pathways exerted robust analgesic effects (33). Although circulating inflammatory markers did not differ significantly between treatment groups, it does not preclude that krill oil may have had localized anti-inflammatory effects within joints (not assessed). Furthermore, krill oil supplementation may have contributed to pain reduction through other non-anti-inflammatory mechanisms. DHA for example has been shown to ameliorate cartilage degradation in a rat adjuvant-induced arthritis model (34). Interestingly, exploratory analyses showed that participants with the greatest amount of inflammation at baseline had significantly greater improvements in knee pain with krill oil relative to placebo, than those with low or medium inflammation at baseline. Other WOMAC knee outcomes were not significantly modulated by inflammatory status.

It is important to consider whether the statistically significant differences in WOMAC knee outcomes were of clinical importance. The minimum clinically important difference (MCID) represents the smallest difference in patient-reported outcomes perceived by patients as important (35–37). Published MCID values for WOMAC knee pain scales vary considerably, ranging between 2 and 30 (36), even when using the same methodology across studies and between different methodologies within studies (37). Greater disease severity (38, 39) and intervention intensity (i.e., surgical compared with nonsurgical) (36) were shown to result in higher MCID values. In the absence of standardized MCID values we compared our results with minimal clinically important improvement (MCII) values developed by Bellamy et al. (40), the same researchers who developed the WOMAC scales. The adjusted means (95% CIs) for the difference in change from baseline between krill oil and placebo in the current study were below the suggested MCII values (40): WOMAC knee pain, 5 (0.3, 10) compared with 9 (6, 12) for MCII; WOMAC knee stiffness, 6 (1, 12) compared with 7 (5, 9) for MCII; and WOMAC knee physical function, 5 (0.1, 9) compared with 6 (3, 9) for MCII (40). However, a large proportion of participants in the krill oil group achieved at least the lower MCII bounds: for knee pain 75% compared with 65% in the placebo group achieved improvement of ≥6 units; for knee stiffness and physical function 77% compared with 63% and 76% compared with 65% achieved improvements of ≥5 and ≥3 units, respectively. In addition, the effect estimate for change in knee pain in the high inflammatory subgroup (20; 95% CI: 10, 31) was more than twice the MCII value (9; 95% CI: 6, 12). Notably, the current study population had less severe knee OA than the MCII population [KL grade of 1–3 compared with 2–4; baseline mean ± SD WOMAC knee pain of 40 ± 16 and 53 ± 24 in the current and MCII populations (40), respectively]. Thus, comparisons with MCII values should be interpreted with caution.

Effects on serum lipids were consistent with a large body of evidence that showed LC ω-3 PUFA treatment does not affect total cholesterol or HDL cholesterol. Although LDL cholesterol may rise to a small extent, the effect seems transient (41, 42), as was observed in the current study. The normo- to borderline hyperlipidemic study population and EPA + DHA dosage <1 g/d may account for the lack of observed hypotriglyceridemic effect in the current trial (43). Most previous krill oil studies did not show hypotriglyceridemic effects (14, 44, 45) and those that did were poorly reported (46) or executed (47).

No safety concerns related to krill oil treatment arose during this study. Differences between groups for a small number of blood hematology and biochemistry parameters were unlikely to be of clinical relevance. Incidence of treatment-related AEs was low and did not differ statistically between groups, and no treatment-related SAEs were reported. Previous clinical trials with krill oil in OA participants did not report treatment-related AEs (16, 17), although AEs were not well reported in these publications. The current trial did not report treatment-related AEs previously reported in fish oil trials (24, 48), including fish-smelling eructation, gastrointestinal disorders (e.g., flatulence and diarrhea), or bleeding/vascular complications.

A strength of the current study is the rigorous study design: blinding and concealment of study treatments were ensured by physical concealment (identical opaque containers with sequential kit numbers and matched appearance of krill oil and placebo supplements) and by using an independently administered treatment allocation system/IVRS. Another strength includes using a study population for whom the intervention is targeted. Participants with appropriately diagnosed mild to moderate knee OA with room to improve in knee pain were recruited, whereas those with severe knee OA were excluded, because the latter population may be beyond the point of improvement with a supplement or may require high doses of NSAIDs that may mask any supplement effect. Similarly, the study included participants with low habitual intake of LC ω-3 PUFAs who were expected to have capacity to improve their ω-3 status. Omega-3 Index was assessed at baseline and follow-up to confirm low intakes and increases in intake over time with krill oil supplementation, respectively, indicating excellent compliance (49). Use of a valid, reliable, and responsive standardized globalized measure (WOMAC Index) to assess knee pain (40), a sufficiently long intervention duration of 6 mo (50), and selection of a dosage that was higher than those of previous krill oil studies, but appropriate for use as a complementary medicine, further contributed to a thorough and rigorous study. The dropout rate (10%) and amount of missing data were relatively small. WOMAC knee pain, however, remains a subjective tool and assessment of nonsubjective markers, e.g., cartilage volume measured by MRI, may have provided supportive information. A measure of localized joint inflammation, e.g., MRI-assessed effusing/synovitis, may have provided insights into mechanisms whereby krill oil reduced OA knee pain (50). *P* values for secondary analyses were not adjusted for multiple outcomes, such that the type I error rate may be inflated above a nominal level of 5% (2-sided), and these results should be interpreted with caution.

In conclusion, the present study provides robust scientific evidence that consumption of 4 g/d of a commercially available krill oil supplement is safe and resulted in modest improvements in knee pain, stiffness, and physical function compared with a placebo. There was insufficient evidence to suggest treatment-related effects with respect to NSAID usage, any of the serum lipids, or inflammatory or safety markers.

## Supplementary Material

nqac125_Supplemental_File

## Data Availability

Data described in the article, code book, and analytic code will be made available upon request pending application and approval.

## Notes

Supported by Swisse Wellness Pty Ltd. The funding source, in collaboration with the research scientists, designed the trial and monitored its implementation, but had no influence over the analysis, reporting, and interpretation of the data.

Supplemental Methods and Supplemental Tables 1–19 are available from the “Supplementary data” link in the online posting of the article and from the same link in the online table of contents at https://academic.oup.com/ajcn/.

Abbreviations used: AE, adverse event; CRP, C-reactive protein; FOCBP, females of child-bearing potential; hsCRP, high-sensitivity C-reactive protein; IA, intraarticular; IM, intramuscular; ITT, intention-to-treat; IVRS, interactive voice response system; KL, Kellgren-Lawrence; LC, long-chain; MCID, minimum clinically important difference; MCII, minimal clinically important improvement; NSAID, nonsteroidal anti-inflammatory drug; OA, osteoarthritis; PRN, pro re nata (Latin for “as needed”); RCT, randomized controlled trial; SAE, serious adverse event; SAP, statistical analysis plan; VAS, visual analog scale; WOMAC, Western Ontario and McMaster Universities Osteoarthritis Index; %E, percentage of total energy intake.
