# Correction to: Stonehouse et al. Krill oil improved osteoarthritic knee pain in adults with mild to moderate knee osteoarthritis: a 6-month multicenter, randomized, double-blind, placebo-controlled trial. Am J Clin Nutr 2022;116:672–85

**DOI:** 10.1093/ajcn/nqac262

**Published:** 2022-11-03

Address correspondence to WS (e-mail: Welma.Stonehouse@csiro.au).

This is a correction to: Stonehouse et al. Krill oil improved osteoarthritic knee pain in adults with mild to moderate knee osteoarthritis: a 6-month multicenter, randomized, double-blind, placebo-controlled trial. Am J Clin Nutr 2022;116:672–85.

In the originally published version of this manuscript, the dose of astaxanthin consumed was reported incorrectly. In the abstract, 0.45 g/day astaxanthin has been corrected to read 0.45 mg/day astaxanthin.

Within the Methods section, under “Investigational Products,” 0.11 g astaxanthin (within 1 g of krill oil) has been corrected to read 0.11 mg astaxanthin. In the same section, 0.45 g astaxanthin (provided by 4 capsules/day of krill oil) has been corrected to read 0.45 mg astaxanthin.

The authors apologize for the error.

