# Supplementary material for: Krill oil improved osteoarthritic knee pain in adults with mild to moderate knee osteoarthritis: a 6-month multicenter, randomized, double-blind, placebo-controlled trial
Source: Am J Clin Nutr. 2022 Jul 26;116(3):672–85. doi: 10.1093/ajcn/nqac125 (PMC9437987; doi:10.1093/ajcn/nqac125)
Supplement: nqac125_Supplemental_File [file nqac125_supplemental_file.docx]

**Krill oil improved osteoarthritic knee pain in adults with mild to moderate knee osteoarthritis: A 6-month multicentre, randomized double-blind, placebo-controlled trial**

Welma Stonehouse et al.

**ONLINE SUPPLEMENTARY MATERIALS**

**TABLE OF CONTENTS**

[ABBREVIATIONS 2](#_Toc99558690)

[SUPPLEMENTARY METHODS 3](#_Toc99558691)

[Changes to methods after commencement of trial 3](#_Toc99558692)

[Study sites 5](#_Toc99558693)

[Participants inclusion and exclusion criteria 5](#_Toc99558694)

[Clinical diagnosis of OA of the knee and knee pain related assessments 8](#_Toc99558695)

[Prohibited medications 9](#_Toc99558696)

[Western Ontario and McMaster Universities Osteoarthritis Index (WOMAC) 9](#_Toc99558697)

[Blood hematology, biochemistry and coagulation parameters 9](#_Toc99558698)

[Assessment of adverse events 10](#_Toc99558699)

[Assessment of prior and concomitant medications 13](#_Toc99558700)

[Data management and monitoring 14](#_Toc99558701)

[Statistical analysis 15](#_Toc99558702)

[Schedule of assessments 15](#_Toc99558703)

[SUPPLEMENTARY RESULTS 17](#_Toc99558704)

[REFERENCES 42](#_Toc99558705)

ABBREVIATIONS

ACR, American College of Rheumatology; AE, Adverse events; ALP, Alkaline phosphatase; ALT, Alanine aminotransferase; aPTT, Activated partial thromboplastin time; AST, Aspartate aminotransferase; BP, Blood pressure; BMI, Body mass index; CRP, C-reactive protein; eCRF, Electronic case report form; FFQ, Food frequency questionnaire; FOCBP, Females of child-bearing potential; GGT, Gamma glutamyl transpeptidase; HB, Hemoglobin; HDL-C, High-density lipoprotein cholesterol; hsCRP, High sensitivity C-reactive Protein; IA, Intraarticular; ICC, Intraclass correlation coefficient; ICH-GCP, International Council on Harmonisation Good Clinical Practice;; IM, Intramuscular; ITT, Intention-to-treat; KL, Kellgren-Lawrence; LC, Long-chain; LD, Lactate dehydrogenase; LDL-C, Low-density lipoprotein cholesterol; MCHC, Mean cell hemoglobin concentration; MCV, Mean cell volume; NSAID, Non-steroidal anti-inflammatory medication; OA, Osteoarthritis; PCV, Packed cell volume; PT, Prothrombin time; PUFA, Polyunsaturated fatty acids; RBC, REDCap, Research Electronic Data Capture; Red blood cell; SAE, Serious adverse event; SUSAR, Suspected unexpected serious adverse reactions; VAS, Visual analog scale; WCC, White cell count; WOMAC, Western Ontario and McMaster Universities Osteoarthritis Index.

SUPPLEMENTARY METHODS

Changes to methods after commencement of trial

| **Former** | **Change** | **Reason for change** | **Date of protocol change** |
| --- | --- | --- | --- |
| Inclusion criteria: Finger prick dried blood spot Omega-3 Index <5% at screening. | Finger prick dried blood spot Omega-3 Index <6% at screening. | To better represent target age and population. The Omega-3 Index is a biomarker of long chain (LC) omega-3 PUFA intake. The objective of the inclusion criteria was to ensure enrolment of participants with low intakes of LC omega-3 PUFA. The original cut-off of <5% was based on an accepted low Omega-3 Index globally. However, some evidence in Australian samples indicate that the average Omega-3 Index may be higher than that of US populations; ranging from 5-6% in Australian samples (1-6) compared to ~4.5% in US populations (7). Furthermore, Omega-3 Index has been shown to increase with age, about 1.5% increase from age 10 to 60 years have been reported (7). Considering that the study population was an older population (40-65 years) and Australians tend to have higher Omega-3 Index levels, the Omega-3 Index cut-off was increased to <6.0%. | 2 May 2018 |
| Inclusion criteria: Finger prick dried blood spot Omega-3 Index <6% at screening | Criteria removed | Despite increasing the Omega-3 Index criteria cut-off to <6% (see above change) this criteria resulted in significant screen failures (~30%) despite relatively low intakes of LC omega-3 PUFA (<500 mg/day) by individuals screened. It was decided to omit the Omega-3 Index criteria because of the mismatch between low LC omega-3 PUFA intakes and high Omega-3 Index levels; uncertainty regarding a suitable cut-off level for an Australian population; and the possibility that even at a higher Omega-3 Index baseline level there is room for improvement of the Omega-3 Index level based on the dosage of LC omega-3 PUFA provided. A dosage of 0.88 g/d of LC omega-3 PUFA was predicted to increase Omega-3 Index with 2.4 % (8) while increases of up to ~12% have been reported in other studies (7). | 23 October 2018 |
| Secondary outcome: | Addition of secondary outcome: non-steroidal anti-inflammatory drug (NSAID) use. | NSAID use during the intervention was identified as an important factor that may affect the primary outcome, knee pain, and for that reason included as a secondary outcome measure. | 28 January 2020 |

Study sites

The clinical trial was conducted at four Australian sites: CSIRO Nutrition and Health Research Clinic, Adelaide, South Australia; University of the Sunshine Coast Clinical Trials Centre, Sippy Downs and Morayfield Health Hub, Queensland and Emeritus Research, Camberwell, Victoria.

Participants inclusion and exclusion criteria

Inclusion criteria: male or female; 40 - 65 years inclusive; clinical diagnosis of osteoarthritis (OA) of the index knee according to American College of Rheumatology (ACR) criteria for the classification of idiopathic OA of the knee; Kellgren-Lawrence (KL) grade 1-3 of the index knee, evidenced by knee x-ray (weight bearing) performed during the screening period; self-reported pain in the index knee on at least 4 days per week for the last 3 months; pain of the index knee between 4 and 8 cm (inclusive) over the 7 days prior to baseline as self-assessed on a 10 cm visual analog scale (VAS); BMI >18.5 kg/m^2^ and <35 kg/m^2^ at baseline; willingness to abstain from use of restricted medications; habitual intake of LC omega-3 PUFA (from food and supplements) of <500 mg/day as assessed using the validated Australian PUFA food frequency questionnaire (FFQ) (9) during the screening period and willingness to maintain a low intake throughout the study (higher intakes of LC omega-3 PUFA required a 3-month washout period); willing to provide written informed consent. Females of both child-bearing potential (FOCBP) and non-child-bearing potential were included in the study. WOCBP were defined as females who have reached menarche and did not meet the criteria for non-child-bearing potential. FOCBP were defined as females who had a hysterectomy, had both ovaries surgically removed, had current documentation of tubal ligation or were post-menopausal (defined as: 1 year without menses, in females >50 years in the absence of another known cause).

Exclusion criteria: severe radiographic knee OA in any knee defined as KL grade >3; ipsilateral hip OA such that it would compromise assessment of knee pain; fibromyalgia, chronic pain syndrome or other concurrent medical or arthritic condition(s) which could interfere with the evaluation of the index knee; history of Reiter's syndrome, rheumatoid arthritis, psoriatic arthritis, ankylosing spondylitis, arthritis associated with inflammatory bowel disease, sarcoidosis, amyloidosis or any other forms of inflammatory arthritis (e.g. gout, pseudogout: gout was excluded unless the participant was on preventative treatment and had not had an attack in the last 12 months); history of or clinical signs and symptoms of infection in the index joint in the last 5 years; knee pain that was not clinically attributable to OA of the knee (e.g., radicular low back pain and hip pain that was referred to the knee that could cause misclassification); pain in any other area of the lower extremities or back that was equal to or greater than the index knee pain (based on self-report); arthroscopy or open knee surgery in the index knee in the previous 12 months or planned for within the duration of the study period; intraarticular (IA) or intramuscular (IM) corticosteroid (investigational or marketed) in any joint within 3 months of screening or oral corticosteroids (investigational or marketed) within 1 month of screening; IA hyaluronic acid (investigational or marketed) in the index knee within 6 months of screening; any other IA intervention or therapy within 3 months of screening; regular use of opioids/opiates within 4 weeks of the baseline visit equivalent to ≥30 mg/day codeine for 5 days or more, unless participant agreed to a washout period of at least 4 weeks prior to baseline; high dose NSAIDs within the last month, defined as the maximum dose recommended or higher for the symptomatic treatment of arthritis pain (e.g. diclofenac ≥150 mg/day, meloxicam ≥15 mg/day, naproxen ≥1,000 mg/day, piroxicam ≥20 mg/day, ibuprofen >2,400 mg/day, celecoxib >200mg/day), unless participant agreed to a washout period of at least 4 weeks prior to baseline; bleeding disorders, taking anticoagulants at the time or received anticoagulants within 28 days of baseline, with the exception of low dose aspirin up to 150 mg/day; regular use of and not prepared to abstain from glucosamine, fish oil, curcumin and other complementary medicines/supplements that may affect the study results (a washout period of 4 weeks applied prior to baseline, except in the case of fish oil where a 3-month washout applied); positive urine dipstick pregnancy test at screening or baseline, currently pregnant and/or breastfeeding; WOCBP who were not using effective methods of contraception and have not been using effective methods of contraception for 14 days prior to baseline and were not willing to use effective methods of contraception throughout the study; history of or known presence of alcohol abuse or illicit drug use; any surgical history, clinically significant conditions (i.e. renal or urological disease, cardiac disease, liver disease, gastrointestinal disease or any other disease) or organ dysfunction that in the opinion of the investigator may have affected the participant’s ability to participate in the study or the study results; hospitalized at the time or any planned hospitalizations during the study or up to 1 month following the last dose of study product that may have affected the participant’s ability to comply with the study in the opinion of the medical investigator; received an investigational drug within 3 months of baseline that in the opinion of the investigator may have affected the participant’s ability to participate in the study or the study results; known or suspected allergies to the investigational products; history of an adverse reaction or known hypersensitivity to seafood or shellfish; hypertension (blood pressure ≥140/90 mmHg) at screening (participants with an elevated blood pressure at screening were included if able to provide a treating doctor letter stating either that they did not have hypertension or that their hypertension had been well controlled for at least 4 weeks).

Clinical diagnosis of OA of the knee and knee pain related assessments

Diagnosis of OA of the knee was made according to clinical diagnosis by a medical investigator using the ACR criteria for the classification of idiopathic OA of the knee (10) and the KL grading scale. Clinical idiopathic OA of the knee was defined as knee pain plus at least 3 of the following 6 criteria: age >50 years; morning stiffness <30 minutes duration; crepitus on active motion; tenderness of the bony margins of the joint; bony enlargement noted on examination; lack of palpable warmth of the synovium (10).

Knee pain was defined as experiencing knee pain on at least 4 days per week for at least 3 months and a VAS-reported knee pain score between 4 and 8 cm, inclusive, on a 10 cm scale for the 7 days prior to baseline.

Severity of OA was assessed using the KL radiographic criteria (11) based on weight bearing knee x-ray. Mild to moderate OA was defined as a KL grade of 1-3 inclusive. Participants with severe radiographic knee OA (KL grade >3) in any knee were excluded. To reduce variation in scoring all x-rays were scored by a single independent radiology practice in Adelaide. Reliability assessments of KL-grades were determined using 20 randomly selected knee x-rays for blinded repeat scoring by the same radiographer (intra-grader reliability) and by a second radiographer (inter-grader reliability). The corresponding intraclass correlation coefficients (ICC) indicated intra-grader reliability ranged from good to excellent (average ICC 0.91, 95%CI [0.79, 0.96]) and inter-grader reliability from moderate to excellent (average ICC 0.83 [0.64, 0.92]), which was consistent with or better than that reported by other similar investigations (12-14).

In cases of bilateral knee OA, both knees were assessed. At least one knee had to meet the defined criteria for study eligibility. The knee with the most severe OA (according to pain score), that met eligibility criteria was chosen as the index knee and only this knee was then followed throughout the clinical study. The index knee was clearly documented in the source documents and explained to the participant.

Prohibited medications

The following medications were prohibited during the study: anticoagulants and antiplatelet medications, except for low dose aspirin up to 150 mg/day; high dose NSAIDs defined as the maximum dose recommended for the symptomatic treatment of arthritis pain or higher (e.g. diclofenac ≥150 mg/day, aceclofenac ≥100 mg/day, meloxicam ≥15 mg/day, naproxen ≥1,000 mg/day, piroxicam ≥20 mg/day, ibuprofen >2,400 mg/day, celecoxib ≥200 mg/day); IM corticosteroids; IA corticosteroids to either knee; any IA intervention or therapy; regular oral corticosteroids (short course ≤10 days of oral corticosteroids were acceptable); other investigational treatments; regular opioids and opiates equivalent to ≥30 mg/day codeine for 5 days or more.

Western Ontario and McMaster Universities Osteoarthritis Index (WOMAC)

The WOMAC Questionnaire comprised the following 24 items, divided into 3 subscales:

Knee pain (5 items): during walking, using stairs, in bed, sitting or lying, and standing

Knee stiffness (2 items): after first waking and later in the day

Knee physical function limitations (17 items): stair use, rising from sitting, standing, bending, walking, getting in / out of a car, shopping, putting on / taking off socks, rising from bed, lying in bed, getting in / out of bath, sitting, getting on / off toilet, heavy household duties, light household duties

Incomplete items were handled according to the WOMAC user guide (15). Accordingly, in the event that one pain, one stiffness or one to three physical function items were missing, the missing values were substituted with the average value of the responses of the corresponding subscale.

Blood hematology, biochemistry and coagulation parameters

The following parameters were assessed:

Hematology: hemoglobin, red blood cell (RBC) count, packed cell volume (PCV), mean cell volume (MCV), mean cell hemoglobin concentration (MCHC), platelets, white cell count (WCC), neutrophils, lymphocytes, monocytes, eosinophils, basophils.

Biochemistry: sodium, potassium, chloride, bicarbonate, glucose, urea, creatinine, calcium, C-reactive protein (CRP), uric acid, phosphate, albumin, globulin, total protein, total bilirubin, gamma glutamyl transpeptidase (GGT), alkaline phosphatase (ALP), alanine aminotransferase (ALT), aspartate aminotransferase (AST), lactate dehyrogenase (LD).

Coagulation: activated partial thromboplastin time (aPTT) and prothrombin time (PT).

Assessment of adverse events

Incidence of adverse event (AE) and serious adverse events (SAE) were recorded from baseline until the final safety visit or early withdrawal. At each clinic visit and online survey participants were questioned in a non-leading manner regarding the occurrence of any AE. For each AE the following information were captured: date of onset, a description of the AE, duration, actions taken, outcome and a medical investigator’s opinion on severity and causality to study treatment. AEs were reported as diagnoses, if available, instead of individual signs and symptoms. Use of all concomitant medications and abnormal laboratory values considered clinically significant by a medical investigator were recorded as an AE.

Definitions

- An AE is any untoward medical occurrence associated with the use of a medicine which does not necessarily have to have a causal relationship with the treatment. An AE can therefore be any unfavourable and unintended sign (including an abnormal laboratory finding), symptom, or disease temporally associated with the use of a medicinal product, whether or not considered related to the medicinal product. This includes any worsening (i.e. any clinically significant adverse change in frequency and/or intensity) of pre-existing condition, which is temporally associated with the use of the study treatment.
- An adverse drug reaction is any AE where a causal relationship with the study product is at least a reasonable possibility (possibly related, probably related or definitely related). ‘Reasonable possibility’ means there is evidence to suggest a causal relationship between the study product and the adverse event.
- A serious adverse event (SAE) or serious adverse reaction is one that:
  - results in death
  - is life-threatening (note: if the participant was an immediate risk of death at the time of the event; it does not refer to an event which hypothetically might have caused death if the event was more severe)
  - requires hospitalization or prolongation of hospitalization
  - results in persistent or significant disability/incapacity
  - may have caused congenital anomaly/birth defect in the offspring of a participant
  - is a medically important event (an event that does not meet any of the other outcomes but may jeopardize the participant and may require medical or surgical intervention (treatment) to prevent one of the other outcomes. Examples include allergic bronchospasm requiring treatment in an emergency room, serious blood dyscrasias or seizures/convulsions that do not result in hospitalization. The development of drug dependence or drug abuse would also be examples of important medical events).

Planned hospital admissions (before or during the study) were not considered SAEs if the illness or disease existed prior to the participant being randomized provided that the condition did not deteriorate in an unexpected way during the study.
SAEs were reported to the Sponsor within 24 hours of becoming aware of the SAE, regardless of causality and HREC were notified of any SAE in accordance with local requirements.

- Suspected unexpected serious adverse reactions (SUSARs) are AEs that are believed to be related to a study treatment and are both unexpected (i.e. the nature or severity is not expected from the information known on the product) and serious. SUSARs require expedited reporting to applicable regulatory authorities.

Coding of AE

All reported AEs/SAEs were coded using the latest version of Medical Dictionary for Regulatory Activities (MedDRA; <https://www.meddra.org> ). The coded items were approved by the principal investigator / medical investigator for each relevant site.

Assessment of Severity

The severity of each AE/SAE was assessed by a medical investigator according to the following categories:

- Mild: An event that is easily tolerated by the participant, causes minimal discomfort, and does not interfere with daily activities.
- Moderate: An event that is sufficiently discomforting to interfere with normal everyday activities; intervention may be required.
- Severe: An event that prevents normal everyday activities; treatment or other intervention is usually needed.

Assessment of Causality

The causality of each AE/SAE was assessed by a medical investigator to determine whether there was a reasonable possibility that the AE may have been caused by the study treatment according to the following categories:

- Not related: The AE is clearly explained by another cause not related to the study product.
- Probably not related: A potential relationship between study product and the AE could exist (i.e. the possibility cannot be excluded), but the AE is most likely explained by causes other than the study agent.
- Possibly related: The AE and administration of study product are reasonably related in time, and the AE can be explained equally well by causes other than the study product.
- Probably related: The AE and use of study product are reasonably related in time, and the AE is more likely explained by study product than other causes.
- Definitely related: The AE and use of study product are related in time, and a direct association can be demonstrated.

Assessment of Outcome

For each recorded AE/SAE the medical investigator assessed the outcome at the time of last observation as follows:

- Fatal: The participant died
- Resolved: The AE or SAE has ended
- Resolved with sequelae: The AE or SAE has ended but changes are noted from baseline
- Unresolved: The AE has not ended. The AE outcome can only be categorized as unresolved if the AE is:
  - Ongoing at the final safety or early withdrawal visit and the medical investigator deems that no further follow-up is required
  - Lost to follow-up after repeated unsuccessful attempts to contact the participant
  - Ongoing and referred to the participant’s physician or specialist

Assessment of prior and concomitant medications

All medications including prescription and non-prescription, supplements and herbal medicines or investigational agents taken by participant 30 days prior to baseline until final safety assessment or early withdrawal were recorded. At each clinic visit and online survey, participants were questioned to assess if they have commenced, stopped or had any changes in concomitant medications since the previous visit, including changes to doses or reason for use. To assist participants with remembering this information they were given a medication checklist to record the information at the time the medications were taken.

Data management and monitoring

Data collected during this study was handled, processed, and managed as per the approved study specific data management plan. All data collected from a participant was recorded in source documents (paper and/or direct to electronic). Study sites entered data from the participants’ source notes into an electronic case report form (eCRF) designed in Research Electronic Data Capture (REDCap), a secure, web-based software platform designed to support data capture for research studies (16, 17).

Copies of all source, eCRF and study related documentation are retained at each site. Final de-identified datasets will be shared with the trial sponsor. Following completion of the study and publication of results, all study related documents and data will be sent to archives and will be retained at least for 15 years.

Study monitoring was performed in accordance with applicable regulations, guidelines, and sponsor procedures. The study monitors verified source documents at regular intervals to ensure that the data collected in the eCRF were accurate and reliable in accordance with ICH-GCP. The following were reviewed at these visits:

- Compliance with the protocol
- Consent procedures
- Source documents
- AE / SAE
- Storage and accountability of materials including Investigational Product
- Investigator Site File

Statistical analysis

Calculating estimates for mean percentage change from estimates in natural logarithm scale:

Estimates for mean percentage change were derived from estimates in natural logarithm scale as follows:

| **Estimate** | **Formula** |
| --- | --- |
| Adjusted mean % change from  baseline | 100 × [exp(Adjusted mean change from baseline in natural logarithm) – 1] |
| Lower 95% confidence limit for adjusted mean % change from baseline | 100 × [exp(lower 95% confidence limit for adjusted mean change from baseline in natural logarithm) – 1] |
| Upper 95% confidence limit for adjusted mean % change from baseline | 100 × [exp(upper 95% confidence limit for adjusted mean change from baseline in natural logarithm) – 1] |
| Difference in adjusted mean % change from baseline (Krill oil relative to Placebo) | 100 × (exp(difference in adjusted mean change from baseline between groups in natural logarithm) – 1) |
| Lower 95% confidence limit for difference in adjusted mean % change from baseline (Krill oil relative to Placebo) | 100 × (exp(lower 95% confidence limit for difference in adjusted mean change from baseline between groups in natural logarithm) – 1) |
| Upper 95% confidence limit for difference in adjusted mean % change from baseline (Krill oil relative to Placebo) | 100 × (exp(upper 95% confidence limit for difference in adjusted mean change from baseline between groups in natural logarithm) – 1) |

Schedule of assessments

See Supplemental Table 1

**SUPPLEMENTAL TABLE 1**

**Schedule of Assessments**

| **Assessment** | | **Visit (day)** | | | | | | | | |
| --- | --- | --- | --- | --- | --- | --- | --- | --- | --- | --- |
|  |  | **Screening**  **(-29 to -1)** | **Baseline**  **(1)** | **1-month**  **(29±3)** | **2-month**  **(57±3)** | **3-month**  **(85±3)** | **4-month**  **(113±3)** | **5-month**  **(141±3)** | **6-month^2^**  **(169±3)** | **Final Safety**  **(197±3)** |
| Online survey | |  |  | X | X |  | X | X |  | X |
| Clinic visits | | X | X |  |  | X |  |  | X |  |
| Informed consent | | X | X |  |  |  |  |  |  |  |
| Demographics | | X |  |  |  |  |  |  |  |  |
| Height and weight | | X | X^3^ |  |  | X^3^ |  |  | X^3^ |  |
| OA assessment: | Self-reported knee pain | X | X |  |  |  |  |  |  |  |
|  | Knee pain VAS | X | X |  |  |  |  |  |  |  |
| Clinical assessment of knee OA | | X | X |  |  |  |  |  |  |  |
| Knee X-ray | | X**^1^** |  |  |  |  |  |  |  |  |
| PUFA FFQ | | X**^1^** |  |  |  |  |  |  |  |  |
| Urine pregnancy test (WOCBP only) | | X | X |  |  | X |  |  | X |  |
| Blood collection (serum): | Hematology, Biochemistry, Coagulation | X | X |  |  | X |  |  | X |  |
|  | Lipids | X | X |  |  | X |  |  | X |  |
|  | Inflammatory markers |  | X |  |  | X |  |  | X |  |
| Vital signs | | X | X |  |  | X |  |  | X |  |
| Medical and surgical history | | X | X |  |  |  |  |  |  |  |
| Physical examination | | X | X |  |  |  |  |  | X^4^ |  |
| Knee WOMAC | |  | X |  |  | X |  |  | X |  |
| Omega-3 Index (finger prick) | |  | X |  |  | X |  |  | X |  |
| Adverse events | |  |  | X | X | X | X | X | X | X |
| Concomitant medications | |  | X | X | X | X | X | X | X | X |
| Treatment randomization | |  | X |  |  |  |  |  |  |  |
| Providing study treatments | |  | X |  |  | X |  |  |  |  |
| Consuming study treatment | |  | X | X | X | X | X | X | X |  |
| Review study treatment compliance | |  |  | X | X | X | X | X | X |  |

PUFA FFQ, polyunsaturated fatty acid food frequency questionnaire; OA, osteoarthritis; VAS, visual analog scale; FOCBP, females of child-bearing potential; WOMAC; Western Ontario and McMaster Universities Osteoarthritis Index
^1^When a screened participant was unable to be enrolled within 28 days, but the time frame did not exceed 3 months from the screen date, and the participant did not change their dietary pattern or supplement intake, PUFA FFQ and Knee x-ray did not have to be repeated; ^2^Or early withdrawal visit; ^3^Weight only; ^4^Symptom directed physical examination

SUPPLEMENTARY RESULTS

SUPPLEMENTAL TABLE 2

**Descriptive statistics for Body mass index (kg/m^2)^**

|  | **Placebo (n=102)** | | **Krill oil (n=104)** | |
| --- | --- | --- | --- | --- |
|  | **Baseline** | **6 months** | **Baseline** | **6 months** |
| Mean (SD) | 28.5 (3.60) | 28.5 (4.00) | 28.1 (3.90) | 28.5 (4.80) |
| Mean (SD) Δ | - | -0.03 (1.44) | - | 0.38 (3.15) |

Δ, change from baseline
Difference in change in BMI between treatment groups from an independent samples t-test was not significant (t(207) = -1.24; p=0.22)

SUPPLEMENTAL TABLE 3

**Participant compliance over the 6-month study period**

|  | **Placebo (n=118)** | **Krill oil (n=117)** |
| --- | --- | --- |
| **Protocol deviations potentially impacting study outcomes:** |  |  |
| No major deviations | 108 (91.5%) | 109 (93.2%) |
| Major deviations^1^ | 10 (8.5%) | 8 (6.8%) |
| **Consumption of study product** |  |  |
| Consumed <80% of study treatment | 19 (16.1%) | 14 (12.0%) |
| Consumed ≥80% of the study treatment | 99 (83.9%) | 103 (88.0%) |
| **Overall compliance** |  |  |
| Not compliant | 24 (20.3%) | 20 (17.1%) |
| Compliant | 94 (79.7%) | 97 (82.9%) |

^1^Due to the use of prohibited medications and therapies; study treatment compliance; and enrolment of a participant with blood pressure levels outside of exclusion criteria limits (participant’s eligibility was revoked after randomization)

SUPPLEMENTAL TABLE 4

**Descriptive statistics for Omega-3 Index (%)**

|  | **Placebo** | | | **Krill oil** | | |
| --- | --- | --- | --- | --- | --- | --- |
|  | **Baseline**  **(n=115)** | **3 months (n=106)** | **6 months (n=103)** | **Baseline (n=116)** | **3 months (n=109)** | **6 months (n=106)** |
| Mean (SD) | 5.5 (0.9) | 5.4 (0.8) | 5.4 (0.8) | 6.0 (1.3) | 8.9 (1.5) | 9.0 (1.6) |
| Mean (SD) Δ | - | -0.06 (0.53) | -0.10 (0.56) | - | 2.89 (1.53) | 2.95 (1.39) |

Δ, change from baseline

**SUPPLEMENTAL TABLE 5**

**Descriptive statistics for WOMAC knee outcomes**

|  | **Placebo** | | | **Krill oil** | | |
| --- | --- | --- | --- | --- | --- | --- |
|  | **Baseline**  **(n=118)** | **3 months**  **(n=106)** | **6 months (n=104)** | **Baseline (n=117)** | **3 months (n=110)** | **6 months (n=106)** |
| **WOMAC knee pain score^1^** | | | | | | |
| Mean (SD) | 39.6 (16.2) | 31 (20.8) | 27.8 (20.6) | 40.6 (15.4) | 29.2 (18.4) | 22.6 (17.8) |
| Mean (SD) Δ | - | -8.6 (18.8) | -12.0 (19.8) | - | -11.8 (16.0) | -18.2 (17.2) |
| **WOMAC knee stiffness score^1^** | | | | | | |
| Mean (SD) | 49.5 (21.0) | 37.0 (23.5) | 38.0 (25.0) | 50.0 (19.5) | 36.5 (22.5) | 29.5 (21.0) |
| Mean (SD) Δ | - | -12.5 (24.5) | -12.0 (26.0) | - | -14.5 (22.0) | -20.5 (22.0) |
| **WOMAC knee physical function score^1^** | | | | | | |
| Mean (SD) | 38.2 (17.7) | 30.4 (21.2) | 28.2 (22.5) | 37.0 (17.2) | 27.1 (18.9) | 22.1 (18.0) |
| Mean (SD) Δ | - | -7.79 (18.2) | -9.85 (21.7) | - | -10.3 (17.1) | -14.8 (17.7) |
| **WOMAC knee total score^1^** | | | | | | |
| Mean (SD) | 39.7 (16.7) | 31.1 (20.7) | 29.1 (21.9) | 39.1 (16.0) | 28.5 (18.6) | 23.0 (17.8) |
| Mean (SD) Δ | - | -8.40 (17.7) | -10.5 (20.9) | - | -11.0 (16.1) | -16.1 (17.0) |

Δ, change from baseline; WOMAC, Western Ontario and McMaster Universities Osteoarthritis Index.
**^1^**WOMAC scores were normalized to scores ranging from 0-100.

**SUPPLEMENTAL TABLE 6**

**Adjusted mean (95% CI) changes in WOMAC knee pain, stiffness, physical function and total scores from baseline at 6 months and comparisons between treatment groups stratified by baseline inflammatory status**

| **Inflammatory status:** | **Placebo**  **n=101** | **Krill oil**  **n=104** | **Krill oil *vs*. Placebo** | **P-value^1^** |
| --- | --- | --- | --- | --- |
| **WOMAC knee pain** | | | | |
| Low [<1 mg/L hsCRP] | -12.4 [-18.6, -6.16] | -16.3 [-21.6, -10.9] | -3.88 [-12.0, 4.24] | 0.35 |
| Medium [≥ 1 mg/L - ≤3 mg/L hsCRP] | -16.1 [-21.3, -11.0] | -15.3 [-20.6, -10.1] | 0.82 [-6.56, 8.20] | 0.83 |
| High [>3 mg/L hsCRP] | -7.24 [-14.16, -0.32] | -27.6 [-36.1, -19.0] | -20.3 [-30.9, -9.74] | <0.001 |
| **WOMAC knee stiffness** | | | | |
| Low [<1 mg/L hsCRP] | -12.6 [-20.3, -4.75] | -19.1 [-25.8, -12.4] | -6.55 [-16.7, 3.62] | 0.21 |
| Medium [≥ 1 mg/L - ≤3 mg/L hsCRP] | -13.8 [-20.3, -7.3] | -19.0 [-25.5, -12.5] | --5.19 [-14.4, 4.06] | 0.27 |
| High [>3 mg/L hsCRP] | -10.3 [-18.9, -1.67] | -22.6 [-13.3, -4.79] | -12.3 [-25.5, 0.91] | 0.07 |
| **WOMAC knee physical function** | | | | |
| Low [<1 mg/L hsCRP] | -8.72 [-15.3, -2.17] | -15.2 [-20.9, -9.59] | -6.50 [-15.1, 2.07] | 0.14 |
| Medium [≥ 1 mg/L - ≤3 mg/L hsCRP] | -12.1 [-17.5, -6.66] | -12.9 [-18.4, -7.37] | -0.77 [-8.54, 7.00] | 0.85 |
| High [>3 mg/L hsCRP] | -5.71 [-13.0, 1.59] | -19.8 [-28.7, -10.8] | -14.1 [-25.3, -2.87] | 0.01 |
| **WOMAC knee total score** | | | | |
| Low [<1 mg/L hsCRP] | -9.79 [-16.2, -3.39] | -15.8 [-21.3, -10.3] | -5.99 [-14.4, 2.39] | 0.16 |
| Medium [≥ 1 mg/L - ≤3 mg/L hsCRP] | -13.1 [-18.4, -7.76] | -13.9 [-19.3, -8.50] | -0.80 [-8.41, 6.80] | 0.84 |
| High [>3 mg/L hsCRP] | -6.38 [-13.5, 0.75] | -21.7 [-30.4, -12.9] | -15.3 [-26.2, -4.37] | <0.01 |

hsCRP, high-sensitivity C-reactive protein; WOMAC, Western Ontario and McMaster Universities Osteoarthritis Index

WOMAC knee scores were normalized to scores ranging from 0-100.
^1^Comparisons between treatment groups were performed using ANCOVA and an intention-to-treat approach (n=205)*; changes from baseline were calculated by subtracting 6-month data from baseline data and compared while controlling for baseline WOMAC knee pain score, baseline level of the respective outcome variable, study site, gender, Omega-3 Index, age, BMI, and osteoarthritis severity. *The analysis population included all randomized participants with a 6-month assessment and complete covariate data. The overall P-values for the treatment x inflammatory status interactions were P=0.01; P=0.68; P=0.16; P=0.10 for knee pain, stiffness, physical function and total scores, respectively.

**SUPPLEMENTAL TABLE 7**

**Adjusted mean (95% CI) changes in WOMAC knee pain score from baseline at 6 months and comparisons between treatment groups stratified for baseline inflammatory status (measured continuously; sensitivity analysis)**

| **Inflammatory status:** | **Placebo**  **n=101** | **Krill oil**  **n=104** | **Krill oil *vs*. Placebo** | **P-value^1^** |
| --- | --- | --- | --- | --- |
| Low [<1 mg/L hsCRP; mean hsCRP 0.57mg/L] | -13.1 [-16.8, -9.5] | -15.9 [-19.7, -12.0] | -2.72 [-8.14, 2.70] | 0.32 |
| Medium [≥ 1 mg/L - ≤3 mg/L hsCRP; mean hsCRP 1.40 mg/L] | -13.0 [-16.5, -9.46] | -16.9 [-20.4, -13.4] | -3.92 [-8.87, 1.14] | 0.13 |
| High [>3 mg/L hsCRP; mean hsCRP 6.88 mg/L] | -12.1 [-15.8, -8.38] | -24.0 [-30.8, -16.9] | -11.8 [-19.7, -3.98] | 0.003 |

hsCRP, high-sensitivity C-reactive protein; WOMAC, Western Ontario and McMaster Universities Osteoarthritis Index

WOMAC knee pain scores were normalized to scores ranging from 0-100. Treatment effect estimates were calculated at the mean level of baseline hsCRP for each baseline inflammatory status group.
^1^Comparisons between treatment groups were performed using ANCOVA and an intention-to-treat approach (n=205)*; changes from baseline were calculated by subtracting 6-month data from baseline data and compared while controlling for baseline WOMAC knee pain score, study site, gender Omega-3 Index, age, BMI, and osteoarthritis severity. *The analysis population included all randomized participants with a 6-month assessment and complete covariate data. The overall P-value for the treatment x inflammatory status (baseline hsCRP measured continuously) interaction was P=0.03.

**SUPPLEMENTAL TABLE 8**

**Descriptive statistics for serum lipid outcomes**

|  | **Placebo** | | | **Krill oil** | | |
| --- | --- | --- | --- | --- | --- | --- |
|  | **Baseline**  **(n=116)** | **3 months (n=109)** | **6 months (n=103)** | **Baseline (n=116)** | **3 months (n=110)** | **6 months (n=107)** |
| **Total cholesterol (mmol/L)** | | | | | | |
| Mean (SD) | 5.9 (1.1) | 5.9 (1.1) | 5.9 (1.2) | 5.9 (1.1) | 6.1 (1.2) | 5.9 (1.2) |
| Mean (SD) Δ | - | 0.01 (0.54) | -0.01 (0.72) | - | 0.16 (0.69) | 0.03 (0.79) |
| **HDL-cholesterol (mmol/)** | | | | | | |
| Mean (SD) | 1.4 (0.3) | 1.4 (0.3) | 1.4 (0.3) | 1.5 (0.4) | 1.5 (0.4) | 1.5 (0.4) |
| Mean (SD) Δ | - | 0.02 (0.20) | 0.02 (0.16) | - | 0.05 (0.14) | 0.05 (0.17) |
| **LDL-cholesterol (mmol/L)** | | | | | | |
| Mean (SD) | 3.8 (1.0) | 3.8 (1.0) | 3.8 (1.1) | 3.8 (0.9) | 4.0 (1.0) | 3.8 (1.1) |
| Mean (SD) Δ | - | -0.02 (0.46) | -0.03 (0.62) | - | 0.17 (0.61) | 0.01 (0.70) |
| **Triglycerides (mmol/L)** | | | | | | |
| Mean (SD) | 1.5 (0.9) | 1.5 (1.1) | 1.5 (0.9) | 1.4 (0.9) | 1.3 (0.8) | 1.4 (0.8) |
| Median (IQR)^1^ | 1.2 (0.9, 1.9) | 1.3 (0.9, 1.8) | 1.2 (0.9, 1.9) | 1.2 (0.9, 1.7) | 1.1 (0.9, 1.6) | 1.2 (0.9, 1.6) |
| Mean (SD) Δ | - | 0.02 (0.86) | -0.00 (0.61) | - | -0.11 (0.53) | -0.06 (0.55) |

Δ, change from baseline; HDL, high-density lipoprotein; LDL, low-density lipoprotein
^1^Median (IQR) are presented as serum triglycerides were not normally distributed

SUPPLEMENTAL TABLE 9

**Descriptive statistics for serum inflammatory outcomes**

|  | **Placebo** | | | **Krill oil** | | | |
| --- | --- | --- | --- | --- | --- | --- | --- |
|  | **Baseline**  **(n=116)** | **3 months (n=109)** | **6 months (n=103)** | **Baseline (n=116)** | **3 months (n=110)** | **6 months (n=107)** | |
| **Interleukin-6 (pg/mL)** | | | | | | |  |
| Median (IQR) | 0.0 (0.0-3.9) | 0.0 (0.0-3.7) | 0.0 (0.0-2.6) | 0.0 (0.0-4.5) | 0.0 (0.0-5.8) | 0.0 (0.0-4.7) | |
| Median (IQR) Δ | - | 0.0 (-0.27, 0.15) | 0.0 (-0.20, 0.0) | - | 0.0 (0.0, 0.67) | 0.0 (0.0, 2.02) | |
| **Tumor necrosis factor-alpha (pg/mL)** | | | | | | |  |
| Median (IQR) | 3.9 (2.2-6.6) | 3.8 (2.8-6.3) | 3.8 (2.7-7.2) | 3.8 (2.2-6.1) | 3.7 (2.3-6.5) | 3.8 (2.0-6.3) | |
| Median (IQR) Δ | - | 0.12 (-1.17, 1.05) | 0.12 (-0.99, 1.12) | - | 0 (-1.08, 1.01) | 0.09 (-0.88, 1.30) | |
| **High sensitivity C-Reactive Protein (mg/L)** | | | | | | |  |
| Median (IQR) | 1.4 (0.8-3.0) | 1.3 (0.9-3.3) | 1.6 (0.8-2.8) | 1.3 (0.7-2.1) | 1.2 (0.7-2.2) | 1.2 (0.7-2.1) | |
| Median (IQR) Δ | - | 0.02 (-0.51, 0.33) | 0.03 (-0.53, 0.33) | - | 0.0 (-0.31, 0.35) | 0.04 (-0.32, 0.22) | |

Δ, change from baseline;
All variables are presented as median (IQR) as they were not normally distributed

SUPPLEMENTAL TABLE 10

**Adjusted mean (95% CI) changes in hsCRP from baseline at 6 months and comparisons between treatment groups stratified by baseline inflammatory status**

| **Inflammatory status:** | **Placebo**  **n=101** | **Krill oil**  **n=104** | **Krill oil *vs*. Placebo** | **P-value^1^** |
| --- | --- | --- | --- | --- |
| Low [<1 mg/L hsCRP; mean hsCRP 0.59 mg/L] | 56.7 [29.1, 90.2] | 33.7 [11.2, 60.7] | -14.7 [-34.5, 11.1] | 0.24 |
| Medium [≥ 1 mg/L - ≤3 mg/L hsCRP; mean hsCRP 1.67 mg/L] | -6.93 [-18.5, 6.24] | -3.69 [-15.7, 10.0] | 3.48 [-14.4, 25.1] | 0.72 |
| High [>3 mg/L hsCRP; mean hsCRP 9.08 mg/L] | -51.2 [-61.1, -38.7] | -35.8 [-50.9, -16.0] | 31.4 [-6.25, 84.2] | 0.11 |

hsCRP, high-sensitivity C-reactive protein; WOMAC, Western Ontario and McMaster Universities Osteoarthritis Index

Treatment effect estimates are calculated at the mean level of baseline hsCRP for each baseline inflammatory status group

^1^Comparisons between treatment groups were performed using ANCOVA and an intention-to-treat approach (n=205)*; changes from baseline were calculated by subtracting 6-month data from baseline data and compared while controlling for baseline WOMAC knee pain score, study site, gender, Omega-3 Index, age, BMI, and osteoarthritis severity. *The analysis population included all randomized participants with a 6-month assessment and complete covariate data. The overall P-value for the treatment x inflammatory status interaction was P=0.07.

**SUPPLEMENTAL** **TABLE 11**

**Descriptive statistics of vital sign outcomes**

|  | **Placebo** | | | **Krill oil** | | |
| --- | --- | --- | --- | --- | --- | --- |
|  | **Baseline (n=118)** | **3 months (n=106)** | **6 months (n=104)** | **Baseline (n=117)** | **3 months (n=110)** | **6 months (n=106)** |
| Systolic BP (mmHg) | 124 (13.4) | 125 (12.7) | 125 (11.9) | 121 (11.2) | 121 (11.4) | 124 (12.1) |
| Diastolic BP (mmHg) | 78.1 (7.9) | 78.0 (8.0) | 79.3 (7.8) | 77.8 (7.3) | 77.2 (6.8) | 79.0 (6.7) |
| Pulse rate (bpm) | 64.6 (9.2) | 66.2 (10.3) | 65.7 (10.2) | 65.0 (8.3) | 65.5 (9.1) | 65.0 (9.3) |
| Respiratory rate (breaths/min) | 15.4 (2.2) | 15.3 (2.0) | 15.0 (1.7) | 15.4 (2.1) | 15.1 (1.9) | 15.3 (2.1) |
| Temperature (°C) | 36.4 (0.3) | 36.4 (0.3) | 36.4 (0.3) | 36.4 (0.3) | 36.3 (0.4) | 36.4 (0.4) |

BP, blood pressure. Values are mean (SD).

**SUPPLEMENTAL** **TABLE 12**

**Adjusted mean (95% CI) changes in vital sign outcomes from baseline and comparisons between treatment groups**

|  | **Placebo**  **n=106** | **Krill oil**  **n=110** | **Krill oil *vs*. Placebo** | **P-value^1^** |
| --- | --- | --- | --- | --- |
| **Systolic BP (mmHg)** | | | | |
| Δ 3 months | 1.16 (-0.39, 2.71) | 0.33 (-1.19, 1.85) | -0.83 (-3.01, 1.36) | 0.46 |
| Δ 6 months | 1.91 (0.35, 3.47) | 3.05 (1.51, 4.59) | 1.14 (-1.07, 3.34) | 0.31 |
| **Diastolic BP (mmHg)** | | | | |
| Δ 3 months | -0.07 (-1.14, 1.00) | -0.42 (-1.47, 0.63) | -0.35 (-1.85, 1.15) | 0.64 |
| Δ 6 months | 1.30 (0.22, 2.37) | 1.30 (0.23, 2.37) | 0.00 (-1.51, 1.52) | 1.00 |
| **Pulse rate (bpm)** | | | | |
| Δ 3 months | 1.82 (0.57, 3.07) | 0.51 (-0.72, 1.74) | -1.31 (-3.06, 0.45) | 0.14 |
| Δ 6 months | 1.04 (-0.22, 2.30) | 0.10 (-1.15, 1.35) | -0.94 (-2.72, 0.83) | 0.30 |
| **Respiratory rate (breaths/min)** | | | | |
| Δ 3 months | -0.19 (-0.53, 0.15) | -0.35 (-0.68, -0.01) | -0.16 (-0.63, 0.32) | 0.51 |
| Δ 6 months | -0.47 (-0.81, -0.13) | -0.17 (-0.51, 0.17) | 0.30 (-0.18, 0.78) | 0.22 |
| **Temperature (°C)** | | | | |
| Δ 3 months | -0.05 (-0.11, 0.02) | -0.09 (-0.15, -0.03) | -0.04 (-0.13, 0.05) | 0.35 |
| Δ 6 months | 0.00 (-0.06, 0.06) | -0.01 (-0.07, 0.05) | -0.01 (-0.10, 0.08) | 0.84 |

Δ, change from baseline; BP, blood pressure
^1^Comparisons between treatment groups were performed using linear mixed effects models on intention-to-treat dataset (n=216); changes from baseline were calculated by subtracting 3- and 6-month data from baseline data and compared while controlling for the baseline level of the respective outcome variable, study site, gender, age and baseline WOMAC knee pain score.

**SUPPLEMENTAL** **TABLE 13**

**Incidence of unsatisfactory assessment of physical examination at 6 months and differences between treatment groups**

|  | **Unsatisfactory assessment at 6 months** | | | | | |
| --- | --- | --- | --- | --- | --- | --- |
|  | **Placebo (n=104)** | | **Krill oil (n=106)** | | **Risk Difference**1 | |
| **Physical examination outcome** | n | % [95% CI]2 | n | % [95% CI]^2^ | % [95% CI] | P-value |
| General appearance | 0 | - | 0 | - | - | - |
| Eyes, ears, nose, mouth and throat | 0 | - | 0 | - | - | - |
| Musculoskeletal | 4 | 3.8 [1.1, 9.6] | 2 | 1.9 [0.2, 6.6] | -2.0 [-6.5, 2.6] | 0.39 |
| Respiratory | 0 | - | 0 | - | - | - |
| Cardiovascular | 0 | - | 0 | - | - | - |
| Gastrointestinal/abdominal | 0 | - | 0 | - | - | - |
| Skin | 0 | - | 0 | - | - | - |
| Kidneys and bladder | 11 | 10.6 [5.4, 18.1] | 7 | 6.7 [2.7, 13.4] | -3.8 [-11.5, 3.8] | 0.32 |

^1^Risk difference was calculated as risk (of unsatisfactory assessment) in Krill oil minus risk in Placebo, expressed as a percentage difference
2Exact binomial confidence interval

**SUPPLEMENTAL** **TABLE 14**

**Descriptive statistics for blood biochemistry, hematology and coagulation outcomes**

|  | **Placebo (n=118)** | | | **Krill oil (n=117)** | | | **Reference Range^2^** |
| --- | --- | --- | --- | --- | --- | --- | --- |
|  | **Baseline** | **3 months** | **6 months** | **Baseline** | **3 months** | **6 months** |  |
| **Serum biochemistry** |  |  |  |  |  |  |  |
| Sodium (mmol/L) | 140 (1.5) | 140 (1.9) | 140 (2.2) | 140 (1.5) | 140 (1.8) | 140 (1.6) | 135-146 |
| Missing, n (%) | 1 (0.8) | 12 (10.2) | 15 (12.7) | 1 (0.9) | 7 (6.0) | 12 (10.3) |  |
| Potassium (mmol/L) | 4.2 (0.3) | 4.2 (0.3) | 4.2 (0.3) | 4.2 (0.3) | 4.2 (0.3) | 4.2 (0.3) | 3.7-5.6 |
| Missing, n (%) | 1 (0.8) | 12 (10.2) | 15 (12.7) | 1 (0.9) | 8 (6.8) | 12 (10.3) |  |
| Chloride (mmol/L) | 104 (2.3) | 105 (2.3) | 105 (2.5) | 104 (2.2) | 104 (2.3) | 104 (2.4) | 96-108 |
| Missing, n (%) | 1 (0.8) | 12 (10.2) | 15 (12.7) | 1 (0.9) | 7 (6.0) | 12 (10.3) |  |
| Bicarbonate (mmol/L) | 28.2 (2.0) | 27.7 (2.1) | 27.7 (2.3) | 28.1 (1.8) | 27.8 (2.4) | 28.1 (2.0) | 20-33 |
| Missing, n (%) | 1 (0.8) | 12 (10.2) | 15 (12.7) | 1 (0.9) | 7 (6.0) | 12 (10.3) |  |
| Glucose (mmol/L) | 5.0 (0.6) | 4.9 (0.7) | 5.0 (0.7) | 4.9 (0.6) | 5.1 (0.7) | 5.1 (0.9) | 3.0-5.4 |
| Missing, n (%) | 1 (0.8) | 12 (10.2) | 15 (12.7) | 0 (0.0) | 7 (6.0) | 12 (10.3) |  |
| Urea (mmol/L) | 5.6 (1.3) | 5.8 (1.4) | 5.7 (1.3) | 5.6 (1.2) | 5.7 (1.2) | 5.6 (1.3) | M: 2.5–10 F: 2.1-10 |
| Missing, n (%) | 1 (0.8) | 12 (10.2) | 15 (12.7) | 1 (0.9) | 7 (6.0) | 12 (10.3) |  |
| Creatinine (μmol/L) | 69.2 (12.9) | 69.3 (13.2) | 69.4 (13.0) | 68.9 (12.3) | 69.9 (12.2) | 68.6 (12.1) | M: 60-110  F: 45-85 |
| Missing, n (%) | 1 (0.8) | 12 (10.2) | 15 (12.7) | 1 (0.9) | 7 (6.0) | 12 (10.3) |  |
| Uric Acid (mmol/L)^1^ | 0.33  (0.27-0.38) | 0.33  (0.28-0.38) | 0.33  (0.27-0.38) | 0.33  (0.28-0.39) | 0.34  (0.29-0.38) | 0.32  (0.28-0.37) | M: 0.19-0.48  F: 0.13-0.43 |
| Missing, n (%) | 2 (1.7) | 12 (10.2) | 15 (12.7) | 1 (0.9) | 7 (6.0) | 12 (10.3) |  |
| Calcium (mmol/L) | 2.3 (0.1) | 2.3 (0.1) | 2.4 (0.1) | 2.4 (0.1) | 2.4 (0.1) | 2.4 (0.1) | 2.10-2.55 |
| Missing, n (%) | 2 (1.7) | 12 (10.2) | 15 (12.7) | 1 (0.9) | 7 (6.0) | 12 (10.3) |  |
| Phosphate (mmol/L) | 1.1 (0.1) | 1.1 (0.2) | 1.1 (0.1) | 1.1 (0.2) | 1.1 (0.2) | 1.1 (0.2) | 0.7-1.5 |
| Missing, n (%) | 2 (1.7) | 12 (10.2) | 15 (12.7) | 1 (0.9) | 8 (6.8) | 12 (10.3) |  |
| Total bilirubin (μmol/L)^1^ | 11.0  (9.0-14.0) | 10.5  (9.0-13.0) | 10.5  (8.0-14.5) | 11.0  (9.0-14.0) | 11.0  (9.0-13.0) | 11.0  (9.0-14.0) | M: 0-20  F: 0-15 |
| Missing, n (%) | 1 (0.8) | 12 (10.2) | 14 (11.9) | 0 (0.0) | 7 (6.0) | 11 (9.4) |  |
| Total protein (g/L) | 70.3 (4.0) | 70.1 (4.0) | 69.5 (3.9) | 70.6 (3.4) | 70.1 (4.0) | 69.1 (3.5) | 62-87 |
| Missing, n (%) | 1 (0.8) | 12 (10.2) | 14 (11.9) | 0 (0.0) | 7 (6.0) | 11 (9.4) |  |
| Albumin (g/L) | 40.4 (3.0) | 40.5 (3.1) | 40.0 (2.6) | 40.6 (2.7) | 40.0 (2.6) | 39.6 (2.6) | 34-50 |
| Missing, n (%) | 1 (0.8) | 12 (10.2) | 14 (11.9) | 0 (0.0) | 7 (6.0) | 12 (10.3) |  |
| Globulin (g/L) | 29.9 (3.4) | 29.6 (3.5) | 29.5 (3.7) | 30.0 (3.5) | 30.1 (3.5) | 29.4 (3.0) | 20-40 |
| Missing, n (%) | 1 (0.8) | 12 (10.2) | 14 (11.9) | 0 (0.0) | 7 (6.0) | 11 (9.4) |  |
| GGT (U/L)^1^ | 22.0  (17.0-36.0) | 22.0  (15.0-37.0) | 22.0  (16.5-34.5) | 21.0  (16.0-33.5) | 21.0  (16.0-34.0) | 21.0  (16.0-33.0) | M: 0-65  F: 0-50 |
| Missing, n (%) | 1 (0.8) | 12 (10.2) | 14 (11.9) | 1 (0.9) | 7 (6.0) | 11 (9.4) |  |
| AST (U/L)^1^ | 24.0  (21.0-29.0) | 25.0  (22.0-28.0) | 24.0  (20.0-30.0) | 24.0  (20.0-28.0) | 24.0  (20.0-30.0) | 22.0  (20.0-28.0) | 0-45 |
| Missing, n (%) | 1 (0.8) | 12 (10.2) | 14 (11.9) | 0 (0.0) | 8 (6.8) | 11 (9.4) |  |
| ALT (U/L)^1^ | 24.0  (18.0-34.0) | 25.0  (18.0-34.0) | 24.0  (17.0-32.5) | 24.0  (19.0-32.0) | 24.0  (19.0-33.0) | 25.5  (20.0-33.0) | 0-50 |
| Missing, n (%) | 1 (0.8) | 12 (10.2) | 14 (11.9) | 0 (0.0) | 7 (6.0) | 11 (9.4) |  |
| ALP (U/L)^1^ | 74.0  (62.0-88.0) | 76.5  (62.0-90.0) | 77.5  (64.0-92.0) | 71.0  (60.0-89.0) | 70.0  (58.0-84.0) | 72.0  (59.0-87.0) | 30-140 |
| Missing, n (%) | 1 (0.8) | 12 (10.2) | 14 (11.9) | 0 (0.0) | 8 (6.8) | 11 (9.4) |  |
| LD (U/L) | 244 (80.0) | 244 (74.8) | 242 (75.5) | 242 (85.8) | 241 (83.7) | 239 (82.3) | 15-280 |
| Missing, n (%) | 1 (0.8) | 12 (10.2) | 15 (12.7) | 1 (0.9) | 8 (6.8) | 13 (11.1) |  |
| C-Reactive Protein |  |  |  |  |  |  | - |
| <5 mg/L, n (%) | 89 (75.4) | 85 (72.0) | 83 (70.3) | 92 (78.6) | 94 (80.3) | 91 (77.8) |  |
| ≥5 mg/L, n (%) | 22 (18.6) | 21 (17.8) | 20 (16.9) | 19 (16.2) | 16 (13.7) | 14 (12.0) |  |
| Missing, n (%) | 7 (5.9) | 12 (10.2) | 15 (12.7) | 6 (5.1) | 7 (6.0) | 12 (10.3) |  |
| **Hematology** |  |  |  |  |  |  |  |
| HB (g/L) | 144.4 (12.4) | 144.2 (12.0) | 143.7 (12.1) | 143.4 (12.5) | 143.6 (12.9) | 143.3 (13.1) | 115-165 |
| Missing, n (%) | 1 (0.8) | 12 (10.2) | 14 (11.9) | 0 (0.0) | 7 (6.0) | 12 (10.3) |  |
| RBC count (x10^12^/L) | 4.8 (0.4) | 4.8 (0.5) | 4.8 (0.4) | 4.8 (0.4) | 4.8 (0.4) | 4.8 (0.4) | M: 4-5-6.5  F: 3.9-5.6 |
| Missing, n (%) | 1 (0.8) | 13 (11.0) | 14 (11.9) | 0 (0.0) | 7 (6.0) | 12 (10.3) |  |
| PCV (%) | 0.44 (0.03) | 0.44 (0.03) | 0.43 (0.03) | 0.43 (0.03) | 0.44 (0.03) | 0.43 (0.03) | M: 0.38-0.52  F: 0.37-0.47 |
| Missing, n (%) | 1 (0.8) | 14 (11.9) | 15 (12.7) | 0 (0.0) | 7 (6.0) | 12 (10.3) |  |
| MCV (fL) | 90.9 (3.9) | 91.0 (4.1) | 91.1 (3.8) | 91.1 (4.6) | 91.1 (4.8) | 91.4 (4.9) | 80-100 |
| Missing, n (%) | 1 (0.8) | 13 (11.0) | 14 (11.9) | 0 (0.0) | 7 (6.0) | 12 (10.3) |  |
| MCHC (g/L) | 331 (9.7) | 330 (11.2) | 331 (10.6) | 330 (11.2) | 330 (9.7) | 330 (10.6) | 310-360 |
| Missing, n (%) | 1 (0.8) | 13 (11.0) | 14 (11.9) | 0 (0.0) | 7 (6.0) | 12 (10.3) |  |
| WCC (x10^9^/L) | 5.7 (1.8) | 5.7 (1.8) | 5.4 (1.3) | 5.6 (1.4) | 5.4 (1.5) | 5.4 (1.4) | 4.0-11.0 |
| Missing, n (%) | 1 (0.8) | 12 (10.2) | 14 (11.9) | 0 (0.0) | 7 (6.0) | 12 (10.3) |  |
| Neutrophils (x10^9^/L)^1^ | 3.1 (2.4-3.9) | 3.0 (2.5-3.7) | 3.0 (2.3-3.5) | 3.0 (2.4-3.7) | 2.7 (2.2-3.5) | 2.6 (2.3-3.5) | 2.0-7.5 |
| Missing, n (%) | 1 (0.8) | 12 (10.2) | 14 (11.9) | 0 (0.0) | 7 (6.0) | 12 (10.3) |  |
| Lymphocytes (x10^9^/L)^1^ | 1.7 (1.5-2.1) | 1.7 (1.5-2.1) | 1.7 (1.5-2.0) | 1.7 (1.5-2.0) | 1.8 (1.5-2.0) | 1.7 (1.4-2.1) | 1.0-4.0 |
| Missing, n (%) | 1 (0.8) | 12 (10.2) | 14 (11.9) | 0 (0.0) | 7 (6.0) | 12 (10.3) |  |
| Platelets (x10^9^/L) | 256 (51.0) | 256 (52.6) | 254 (51.6) | 255 (51.6) | 254 (51.6) | 256 (55.6) | 150-400 |
| Missing, n (%) | 1 (0.8) | 12 (10.2) | 14 (11.9) | 0 (0.0) | 7 (6.0) | 12 (10.3) |  |
| Monocytes (x10^9^/L) | 0.45 (0.14) | 0.45 (0.13) | 0.44 (0.13) | 0.47 (0.14) | 0.43 (0.14) | 0.42 (0.13) | 0.2-1.0 |
| Missing, n (%) | 1 (0.8) | 12 (10.2) | 14 (11.9) | 0 (0.0) | 7 (6.0) | 12 (10.3) |  |
| Eosinophils (x10^9^/L) | 0.15 (0.10) | 0.16 (0.10) | 0.15 (0.09) | 0.16 (0.12) | 0.15 (0.10) | 0.15 (0.10) | 0-0.5 |
| Missing, n (%) | 1 (0.8) | 12 (10.2) | 14 (11.9) | 0 (0.0) | 7 (6.0) | 12 (10.3) |  |
| Basophils (x10^9^/L) | 0.06 (0.05) | 0.05 (0.05) | 0.05 (0.05) | 0.06 (0.05) | 0.05 (0.05) | 0.05 (0.05) | 0-0.1 |
| Missing, n (%) | 1 (0.8) | 12 (10.2) | 14 (11.9) | 0 (0.0) | 7 (6.0) | 12 (10.3) |  |
| **Coagulation** |  |  |  |  |  |  |  |
| aPTT (sec) | 28.7 (2.3) | 28.7 (2.3) | 29.0 (2.0) | 28.5 (2.2) | 28.6 (2.2) | 28.9 (2.2) | 24-34 |
| Missing, n (%) | 2 (1.7) | 12 (10.2) | 14 (11.9) | 1 (0.9) | 9 (7.7) | 12 (10.3) |  |
| PT (sec) | 12.0 (0.6) | 11.9 (0.7) | 12.0 (0.6) | 12.0 (0.6) | 11.8 (0.7) | 11.9 (1.2) | 8.0-14.0 |
| Missing, n (%) | 3 (2.5) | 12 (10.2) | 14 (11.9) | 2 (1.7) | 9 (7.7) | 11 (9.4) |  |

ALP, alkaline phosphatase; ALT, alanine aminotransferase, aPPT, activated partial thromboplastin time; AST, aspartate aminotransferase; F, female; GGT, gamma glutamyl transpeptidase; HB, hemoglobin; LD, lactate dehydrogenase; M, male; MCHC, mean cell haemoglobin concentration; MCV, mean cell volume; PCV, packed cell volume; PT, prothrombin time; RBC, red blood cell; WCC, white cell count
Values are presented as mean (SD), ^1^median (IQR) or n (%)
^2^Reference ranges were obtained from Abbott Pathology, Adelaide, South Australia, Australia.

**SUPPLEMENTAL** **TABLE 15**

**Adjusted mean (95% CI) changes in blood biochemistry, hematology and coagulation outcomes from baseline and comparisons between treatment groups**

|  | **Placebo** | **Krill oil** | **Krill oil *vs*. Placebo** | **P-value^1^** |
| --- | --- | --- | --- | --- |
| **Sodium (mmol/L)** | n=106 | n=109 |  |  |
| Δ 3 months | 0.03 [-0.29, 0.34] | 0.22 [-0.10, 0.53] | 0.19 [-0.26, 0.63] | 0.41 |
| Δ 6 months | 0.10 [-0.22, 0.42] | -0.08 [-0.40, 0.24] | -0.18 [-0.63, 0.28] | 0.44 |
| **Potassium (mmol/L)** | n=106 | n=109 |  |  |
| Δ 3 months | -0.05 [-0.10, -0.00] | -0.03 [-0.08, 0.01] | 0.02 [-0.05, 0.09] | 0.56 |
| Δ 6 months | -0.00 [-0.05, 0.05] | -0.04 [-0.09, 0.01] | -0.04 [-0.11, 0.03] | 0.27 |
| **Chloride (mmol/L)** | n=106 | n=109 |  |  |
| Δ 3 months | 0.54 [0.20, 0.88] | 0.31 [-0.02, 0.64] | -0.23 [-0.71, 0.24] | 0.34 |
| Δ 6 months | 0.46 [0.12, 0.80] | 0.35 [0.01, 0.69] | -0.11 [-0.60, 0.37] | 0.64 |
| **Bicarbonate (mmol/L)** | n=106 | n=109 |  |  |
| Δ 3 months | -0.63 [-0.98, -0.28] | -0.41 [-0.76, -0.07] | 0.21 [-0.28, 0.71] | 0.40 |
| Δ 6 months | -0.60 [-0.96, -0.25] | -0.08 [-0.44, 0.27] | 0.52 [0.02, 1.02] | 0.04 |
| **Glucose (mmol/L)** | n=106 | n=109 |  |  |
| Δ 3 months | -0.02 [-0.14, 0.09] | 0.20 [0.09, 0.31] | 0.22 [0.06, 0.38] | 0.01 |
| Δ 6 months | -0.01 [-0.13, 0.10] | 0.25 [0.14, 0.37] | 0.26 [0.10, 0.43] | 0.001 |
| **Urea (mmol/L)** | n=106 | n=109 |  |  |
| Δ 3 months | 0.23 [0.03, 0.42] | 0.13 [-0.06, 0.32] | -0.10 [-0.37, 0.18] | 0.49 |
| Δ 6 months | 0.17 [-0.03, 0.36] | 0.05 [-0.15, 0.24] | -0.12 [-0.40, 0.16] | 0.39 |
| **Creatinine (μmol/L)** | n=106 | n=109 |  |  |
| Δ 3 months | -0.03 [-1.20, 1.13] | 0.74 [-0.41, 1.89] | 0.78 [-0.87, 2.42] | 0.35 |
| Δ 6 months | 0.28 [-0.90, 1.46] | -0.32 [-1.49, 0.85] | -0.60 [-2.26, 1.07] | 0.48 |
| **Uric acid (%)^2^** | n=105 | n=109 |  |  |
| Δ 3 months | 1.54 [-1.07, 4.22] | 2.58 [-0.01, 5.23] | 1.02 [-2.61, 4.78] | 0.59 |
| Δ 6 months | 2.85 [0.17, 5.60] | -2.14 [-4.64, 0.44] | -4.85 [-8.31, -1.26] | 0.01 |
| **Calcium (mmol/L)** | n=105 | n=109 |  |  |
| Δ 3 months | -0.01 [-0.02, 0.01] | -0.00 [-0.02, 0.01] | 0.00 [-0.02, 0.02] | 0.72 |
| Δ 6 months | 0.00 [-0.01, 0.02] | 0.01 [-0.00, 0.02] | 0.01 [-0.01, 0.03] | 0.40 |
| **Phosphate (mmol/L)** | n=105 | n=109 |  |  |
| Δ 3 months | -0.01 [-0.03, 0.02] | 0.00 [-0.02, 0.02] | 0.01 [-0.03, 0.04] | 0.70 |
| Δ 6 months | -0.02 [-0.04, 0.01] | 0.02 [-0.00, 0.05] | 0.04 [0.01, 0.07] | 0.02 |
| **Total bilirubin (%)^2^** | n=106 | n=110 |  |  |
| Δ 3 months | -4.45 [-9.18, 0.53] | -0.74 [-5.57, 4.33] | 3.88 [-3.26, 11.54] | 0.30 |
| Δ 6 months | -2.82 [-7.67, 2.29] | -1.37 [-6.24, 3.75] | 1.49 [-5.56, 9.07] | 0.69 |
| **Total protein (g/L)** | n=106 | n=110 |  |  |
| Δ 3 months | -0.35 [-0.92, 0.22] | -0.42 [-0.98, 0.14] | -0.07 [-0.87, 0.73] | 0.87 |
| Δ 6 months | -0.97 [-1.54, -0.39] | -1.36 [-1.93, -0.79] | -0.39 [-1.20, 0.42] | 0.34 |
| **Albumin (g/L)** | n=106 | n=110 |  |  |
| Δ 3 months | 0.09 [-0.27, 0.44] | -0.59 [-0.94, -0.24] | -0.68 [-1.18, -0.18] | 0.01 |
| Δ 6 months | -0.39 [-0.75, -0.03] | -0.95 [-1.31, -0.60] | -0.57 [-1.07, -0.06] | 0.03 |
| **Globulin (g/L)** | n=106 | n=110 |  |  |
| Δ 3 months | -0.49 [-0.93, -0.04] | 0.17 [-0.27, 0.61] | 0.66 [0.03, 1.28] | 0.04 |
| Δ 6 months | -0.60 [-1.05, -0.15] | -0.42 [-0.87, 0.02] | 0.18 [-0.45, 0.81] | 0.58 |
| **AST (%)^2^** | n=106 | n=110 |  |  |
| Δ 3 months | 0.11 [-4.02, 4.42] | -1.37 [-5.39, 2.81] | -1.48 [-7.15, 4.53] | 0.62 |
| Δ 6 months | -0.07 [-4.23, 4.27] | -0.25 [-4.35, 4.03] | -0.18 [-5.97, 5.97] | 0.95 |
| **ALT (%)^2^** | n=106 | n=110 |  |  |
| Δ 3 months | -2.51 [-7.26, 2.49] | 0.45 [-4.36, 5.50] | 3.04 [-3.93, 10.52] | 0.40 |
| Δ 6 months | -2.79 [-7.56, 2.23] | 5.70 [0.58, 11.09] | 8.74 [1.31, 16.71] | 0.02 |
| **ALP (%)^2^** | n=106 | n=110 |  |  |
| Δ 3 months | 1.23 [-2.10, 4.67] | -1.77 [-4.96, 1.52] | -2.97 [-7.42, 1.70] | 0.21 |
| Δ 6 months | 2.27 [-1.12, 5.78] | 1.63 [-1.71, 5.08] | -0.63 [-5.24, 4.21] | 0.79 |
| **GGT (%)^2^** | n=106 | n=109 |  |  |
| Δ 3 months | -2.91 [-8.71, 3.26] | -0.58 [-6.45, 5.65] | 2.40 [-6.11, 11.68] | 0.59 |
| Δ 6 months | -0.09 [-6.11, 6.32] | 6.20 [-0.17, 12.97] | 6.29 [-2.65, 16.05] | 0.17 |
| **LD (U/L)** | n=106 | n=109 |  |  |
| Δ 3 months | -1.44 [-6.35, 3.48] | -2.77 [-7.64, 2.10] | -1.33 [-8.25, 5.59] | 0.71 |
| Δ 6 months | -1.58 [-6.56, 3.39] | -1.58 [-6.55, 3.39] | 0.00 [-7.03, 7.04] | 1.00 |
| **HB (g/L)** | n=106 | n=110 |  |  |
| Δ 3 months | -0.20 [-1.26, 0.85] | -0.18 [-1.21, 0.86] | 0.03 [-1.45, 1.51] | 0.97 |
| Δ 6 months | -0.66 [-1.72, 0.40] | -0.56 [-1.61, 0.50] | 0.10 [-1.40, 1.60] | 0.89 |
| **RBC count** (x10^12^/L) | n=106 | n=110 |  |  |
| Δ 3 months | 0.04 [-0.01, 0.09] | 0.00 [-0.04, 0.05] | -0.04 [-0.10, 0.03] | 0.28 |
| Δ 6 months | -0.02 [-0.07, 0.03] | -0.03 [-0.07, 0.02] | -0.01 [-0.07, 0.06] | 0.85 |
| **PCV (%)** | n=106 | n=110 |  |  |
| Δ 3 months | 0.00 [-0.00, 0.00] | -0.00 [-0.00, 0.00] | -0.00 [-0.01, 0.00] | 0.56 |
| Δ 6 months | -0.00 [-0.01, 0.00] | -0.00 [-0.01, 0.00] | 0.00 [-0.00, 0.01] | 0.81 |
| **MCV (fL)** | n=106 | n=110 |  |  |
| Δ 3 months | 0.24 [-0.16, 0.63] | -0.01 [-0.40, 0.38] | -0.25 [-0.80, 0.31] | 0.38 |
| Δ 6 months | 0.27 [-0.13, 0.66] | 0.24 [-0.15, 0.63] | -0.03 [-0.59, 0.53] | 0.92 |
| **MCHC (g/L)** | n=106 | n=110 |  |  |
| Δ 3 months | -0.93 [-2.40, 0.55] | -0.04 [-1.49, 1.40] | 0.88 [-1.18, 2.95] | 0.40 |
| Δ 6 months | 0.13 [-1.36, 1.61] | -0.51 [-1.98, 0.97] | -0.64 [-2.73, 1.46] | 0.55 |
| **Platelets (x10^9^/L)** | n=106 | n=110 |  |  |
| Δ 3 months | -2.05 [-6.87, 2.77] | -0.48 [-5.22, 4.25] | 1.57 [-5.20, 8.33] | 0.65 |
| Δ 6 months | -4.04 [-8.90, 0.81] | 2.40 [-2.41, 7.21] | 6.44 [-0.40, 13.29] | 0.07 |
| **WCC (x10^9^/L)** | n=106 | n=110 |  |  |
| Δ 3 months | -0.05 [-0.21, 0.10] | -0.21 [-0.36, -0.06] | -0.16 [-0.37, 0.06] | 0.16 |
| Δ 6 months | -0.37 [-0.52, -0.21] | -0.22 [-0.38, -0.07] | 0.14 [-0.08, 0.36] | 0.20 |
| **Lymphocytes (%)^2^** | n=106 | n=110 |  |  |
| Δ 3 months | -2.05 [-4.81, 0.79] | 0.70 [-2.09, 3.56] | 2.80 [-1.24, 7.01] | 0.18 |
| Δ 6 months | -4.33 [-7.04, -1.53] | -0.41 [-3.22, 2.47] | 4.09 [-0.06, 8.40] | 0.05 |
| **Neutrophils (%)^2^** | n=106 | n=110 |  |  |
| Δ 3 months | -2.17 [-6.19, 2.03] | -6.04 [-9.84, -2.09] | -3.96 [-9.46, 1.86] | 0.18 |
| Δ 6 months | -6.98 [-10.84, -2.96] | -6.17 [-10.04, -2.14] | 0.87 [-4.97, 7.08] | 0.78 |
| **aPTT (sec)** | n=106 | n=109 |  |  |
| Δ 3 months | -0.08 [-0.36, 0.20] | 0.11 [-0.17, 0.38] | 0.19 [-0.20, 0.58] | 0.34 |
| Δ 6 months | 0.21 [-0.07, 0.49] | 0.39 [0.12, 0.67] | 0.19 [-0.21, 0.58] | 0.35 |
| **PT (sec)** | n=106 | n=108 |  |  |
| Δ 3 months | -0.02 [-0.16, 0.12] | -0.11 [-0.25, 0.03] | -0.09 [-0.29, 0.10] | 0.35 |
| Δ 6 months | 0.08 [-0.06, 0.22] | -0.11 [-0.25, 0.03] | -0.19 [-0.39, 0.01] | 0.06 |

Δ, change from baseline; ALP, alkaline phosphatase; ALT, alanine aminotransferase, aPPT, activated partial thromboplastin time; AST, aspartate aminotransferase; F, female; GGT, gamma glutamyl transpeptidase; HB, hemoglobin; LD, lactate dehydrogenase; M, male; MCHC, mean cell hemoglobin concentration; MCV, mean cell volume; PCV, packed cell volume; PT, prothrombin time; RBC, red blood cell; WCC, white cell count
^1^Comparisons between treatment groups were performed using linear mixed effects models on the intention-to-treat dataset; changes from baseline were calculated by subtracting 3- and 6-month data from baseline data and compared while controlling for the baseline level of the respective outcome variable, study site, gender, age and baseline WOMAC knee pain score.
^2^Statistical analyses were performed on log-transformed data; adjusted mean [95% CI] log-data were back-transformed and are presented as mean [95% CI] percentage values.

**SUPPLEMENTAL** **TABLE 16**

**Incidence of adverse events and serious adverse events during the study per treatment group**

|  | **Placebo (n=118)** | **Krill oil (n=117** |
| --- | --- | --- |
| Any adverse event (n, %) | 79 (66.9) | 76 (65.0) |
| Any treatment-related adverse event (n, %) | 11 (9.3) | 9 (7.7) |
| Any serious adverse event (n, %) | 2 (1.7) | 2 (1.7) |
| Any treatment-related serious adverse event (n, %) | 0 | 0 |

**SUPPLEMENTAL** **TABLE 17**

**Summary of adverse events during the study per treatment group (n, %)**

| **Adverse Event^1^** | **Placebo (n=118)** | | | | **Krill oil (n=117)** | | | |
| --- | --- | --- | --- | --- | --- | --- | --- | --- |
|  | None | Mild | Moderate | Severe | None | Mild | Moderate | Severe |
| **Respiratory, thoracic and mediastinal** |  |  |  |  |  |  |  |  |
| Upper respiratory tract infection | 105 (89.0) | 10 (8.5) | 3 (2.5) | 0 (0) | 99 (84.6) | 13 (11.1) | 5 (4.3) | 0 (0) |
| Nasopharyngitis | 108 (91.5) | 7 (5.9) | 3 (2.5) | 0 (0) | 106 (90.6) | 10 (8.5) | 1 (0.9) | 0 (0) |
| Viral upper respiratory tract infection | 113 (95.8) | 4 (3.4) | 1 (0.8) | 0 (0) | 115 (98.3) | 2 (1.7) | 0 (0) | 0 (0) |
| Oropharyngeal pain | 117 (99.2) | 1 (0.8) | 0 (0) | 0 (0) | 114 (97.4) | 3 (2.6) | 0 (0) | 0 (0) |
| Rhinorrhea | 115 (97.5) | 3 (2.5) | 0 (0) | 0 (0) | 115 (98.3) | 2 (1.7) | 0 (0) | 0 (0) |
| Sinusitis | 117 (99.2) | 1 (0.8) | 0 (0) | 0 (0) | 114 (97.4) | 1 (0.9) | 2 (1.7) | 0 (0) |
| **Musculoskeletal and connective tissue disorders** |  |  |  |  |  |  |  |  |
| Arthralgia | 108 (91.5) | 5 (4.2) | 4 (3.4) | 1 (0.8) | 110 (94.0) | 3 (2.6) | 3 (2.6) | 1 (0.9) |
| Back pain | 112 (94.9) | 4 (3.4) | 2 (1.7) | 0 (0) | 110 (94.0) | 2 (1.7) | 4 (3.4) | 1 (0.9) |
| Osteoarthritis | 115 (97.5) | 0 (0) | 3 (2.5) | 0 (0) | 115 (97.9) | 0 (0) | 2 (2.1) | 0 (0) |
| Pain in extremity | 118 (100) | 0 (0) | 0 (0) | 0 (0) | 114 (97.4) | 3 (2.6) | 0 (0) | 0 (0) |
| Neck pain | 117 (99.2) | 1 (0.8) | 0 (0) | 0 (0) | 115 (98.3) | 1 (0.9) | 1 (0.9) | 0 (0) |
| **Gastrointestinal disorders** |  |  |  |  |  |  |  |  |
| Diarrhea | 112 (94.9) | 4 (3.4) | 2 (1.7) | 0 (0) | 112 (95.7) | 4 (3.4) | 0 (0) | 1 (0.9) |
| Gastroenteritis | 115 (97.5) | 1 (0.8) | 1 (0.8) | 1 (0.8) | 115 (98.3) | 1 (0.9) | 1 (0.9) | 0 (0) |
| Nausea | 115 (97.5) | 2 (1.7) | 1 (0.8) | 0 (0) | 114 (97.4) | 2 (1.7) | 1 (0.9) | 0 (0) |
| Gastroesophageal reflux disease | 116 (98.3) | 2 (1.7) | 0 (0) | 0 (0) | 114 (97.4) | 2 (1.7) | 1 (0.9) | 0 (0) |
| Abdominal discomfort | 115 (97.5) | 1 (0.8) | 2 (1.7) | 0 (0) | 116 (99.1) | 1 (0.9) | 0 (0) | 0 (0) |
| Dyspepsia | 116 (98.3) | 2 (1.7) | 0 (0) | 0 (0) | 116 (99.1) | 1 (0.9) | 0 (0) | 0 (0) |
| Vomiting | 116 (98.3) | 0 (0) | 2 (1.7) | 0 (0) | 116 (99.1) | 1 (0.9) | 0 (0) | 0 (0) |
| **Nervous system disorders** |  |  |  |  |  |  |  |  |
| Headache | 101 (85.6) | 12 (10.2) | 4 (3.4) | 1 (0.8) | 110 (94.0) | 7 (6.0) | 0 (0) | 0 (0) |
| Paresthesia | 117 (99.2) | 1 (0.8) | 0 (0) | 0 (0) | 115 (98.3) | 1 (0.9) | 1 (0.9) | 0 (0) |
| **Injury, poisoning and procedural complications** |  |  |  |  |  |  |  |  |
| Contusion | 115 (97.5) | 2 (1.7) | 1 (0.8) | 0 (0) | 115 (98.3) | 1 (0.9) | 1 (0.9) | 0 (0) |
| Ligament sprain | 115 (97.5) | 1 (0.8) | 1 (0.8) | 1 (0.8) | 117 (100) | 0 (0) | 0 (0) | 0 (0) |
| **Infections and infestations** |  |  |  |  |  |  |  |  |
| Oral herpes | 117 (99.2) | 1 (0.8) | 0 (0) | 0 (0) | 115 (98.3) | 2 (1.7) | 0 (0) | 0 (0) |
| **Vascular disorders** |  |  |  |  |  |  |  |  |
| Hypertension | 116 (98.3) | 0 (0) | 2 (1.7) | 0 (0) | 115 (98.3) | 0 (0) | 2 (1.7) | 0 (0) |
| **Immune system disorders** |  |  |  |  |  |  |  |  |
| Seasonal allergy | 116 (98.3) | 2 (1.7) | 0 (0) | 0 (0) | 115 (98.3) | 1 (0.9) | 1 (0.9) | 0 (0) |
| **Psychiatric disorders** |  |  |  |  |  |  |  |  |
| Insomnia | 117 (99.2) | 1 (0.8) | 0 (0) | 0 (0) | 115 (98.3) | 2 (1.7) | 0 (0) | 0 (0) |
| **Metabolism and nutrition disorders** |  |  |  |  |  |  |  |  |
| Hypercholesterolemia | 116 (98.3) | 1 (0.8) | 1 (0.8) | 0 (0) | 115 (98.3) | 2 (1.7) | 0 (0) | 0 (0) |

^1^For each adverse event type, each participant was classified according to the worst grade of severity experienced over the study period and follow-up period. Only those adverse events for which there were 3 or more instances recorded are listed.
Statistical analysis of differences in risk between groups was not undertaken because of low numbers of adverse events reported

**SUPPLEMENTAL** **TABLE 18**

**Summary of treatment-related adverse events during the study per treatment group (n, %)**

| **Treatment related adverse event^1^** | **Placebo (n=118)** | | | | **Krill oil (n=117)** | | | |
| --- | --- | --- | --- | --- | --- | --- | --- | --- |
|  | None | Mild | Moderate | Severe | None | Mild | Moderate | Severe |
| **Gastrointestinal disorders** |  |  |  |  |  |  |  |  |
| Diarrhea | 117 (99.2) | 0 (0) | 1 (0.8) | 0 (0) | 115 (98.3) | 1 (0.9) | 1 (0.9) | 0 (0) |
| Nausea | 117 (99.2) | 1 (0.8) | 0 (0) | 0 (0) | 115 (98.3) | 2 (1.7) | 0 (0) | 0 (0) |
| Abdominal discomfort | 116 (98.3) | 1 (0.8) | 1 (0.8) | 0 (0) | 116 (99.1) | 1 (0.9) | 0 (0) | 0 (0) |
| Dyspepsia | 117 (99.2) | 1 (0.8) | 0 (0) | 0 (0) | 117 (100) | 0 (0) | 0 (0) | 0 (0) |
| Abdominal distension | 117 (99.2) | 1 (0.8) | 0 (0) | 0 (0) | 117 (100) | 0 (0) | 0 (0) | 0 (0) |
| Abdominal pain upper | 117 (99.2) | 0 (0) | 1 (0.8) | 0 (0) | 117 (100) | 0 (0) | 0 (0) | 0 (0) |
| Constipation | 117 (99.2) | 0 (0) | 1 (0.8) | 0 (0) | 117 (100) | 0 (0) | 0 (0) | 0 (0) |
| Frequent bowel movements | 117 (99.2) | 1 (0.8) | 0 (0) | 0 (0) | 117 (100) | 0 (0) | 0 (0) | 0 (0) |
| Gastroesophageal reflux disease | 118 (100) | 0 (0) | 0 (0) | 0 (0) | 116 (99.1) | 1 (0.9) | 0 (0) | 0 (0) |
| Irritable bowel syndrome | 117 (99.2) | 0 (0) | 1 (0.8) | 0 (0) | 117 (100) | 0 (0) | 0 (0) | 0 (0) |
| **Renal and urinary disorders** |  |  |  |  |  |  |  |  |
| Urine odour abnormal | 117 (99.2) | 1 (0.8) | 0 (0) | 0 (0) | 116 (99.1) | 1 (0.9) | 0 (0) | 0 (0) |
| **Skin and subcutaneous tissue disorders** |  |  |  |  |  |  |  |  |
| Seborrhea | 117 (99.2) | 1 (0.8) | 0 (0) | 0 (0) | 117 (100) | 0 (0) | 0 (0) | 0 (0) |
| Vessel puncture site bruise | 118 (100) | 0 (0) | 0 (0) | 0 (0) | 116 (99.1) | 1 (0.9) | 0 (0) | 0 (0) |
| **Eye disorders** |  |  |  |  |  |  |  |  |
| Eye pruritus | 118 (100) | 0 (0) | 0 (0) | 0 (0) | 116 (99.1) | 1 (0.9) | 0 (0) | 0 (0) |
| **Immune system disorders** |  |  |  |  |  |  |  |  |
| Urticaria | 118 (100) | 0 (0) | 0 (0) | 0 (0) | 116 (99.1) | 0 (0) | 1 (0.9) | 0 (0) |
| **Injury, poisoning and procedural complications** |  |  |  |  |  |  |  |  |
| Contusion | 117 (99.2) | 1 (0.8) | 0 (0) | 0 (0) | 117 (100) | 0 (0) | 0 (0) | 0 (0) |
| **Musculoskeletal and connective tissue disorders** |  |  |  |  |  |  |  |  |
| Arthralgia | 117 (99.2) | 1 (0.8) | 0 (0) | 0 (0) | 117 (100) | 0 (0) | 0 (0) | 0 (0) |
| **Nervous system disorders** |  |  |  |  |  |  |  |  |
| Headache | 117 (99.2) | 0 (0) | 0 (0) | 1 (0.8) | 117 (100) | 0 (0) | 0 (0) | 0 (0) |
| **Psychiatric disorders** |  |  |  |  |  |  |  |  |
| Insomnia | 118 (100) | 0 (0) | 0 (0) | 0 (0) | 116 (99.1) | 1 (0.9) | 0 (0) | 0 (0) |
| **Reproductive system and breast disorders** |  |  |  |  |  |  |  |  |
| Hot flush | 117 (99.2) | 1 (0.8) | 0 (0) | 0 (0) | 117 (100) | 0 (0) | 0 (0) | 0 (0) |
| **Respiratory, thoracic and mediastinal disorders** |  |  |  |  |  |  |  |  |
| Throat tightness | 118 (100) | 0 (0) | 0 (0) | 0 (0) | 116 (99.1) | 0 (0) | 1 (0.9) | 0 (0) |
| **Vascular disorders** |  |  |  |  |  |  |  |  |
| Hemorrhage | 118 (100) | 0 (0) | 0 (0) | 0 (0) | 116 (99.1) | 1 (0.9) | 0 (0) | 0 (0) |

^1^For each adverse event type, each participant was classified according to the worst grade of severity experienced over the study period and follow-up period.
Statistical analysis of differences in risk between groups was not undertaken because of low numbers of treatment-related adverse events reported

**SUPPLEMENTAL** **TABLE 19**

**Summary of serious adverse events during the study per treatment group (n, %)**

| **Serious Adverse Event** | **Placebo**  (n=118) | | | | **Krill oil**  (n=117) | | | |
| --- | --- | --- | --- | --- | --- | --- | --- | --- |
|  | None | Mild | Moderate | Severe | None | Mild | Moderate | Severe |
| Bipolar disorder | 118 (100) | 0 (0) | 0 (0) | 0 (0) | 116 (99.1) | 0 (0) | 0 (0) | 1 (0.9) |
| Hepatic cancer | 118 (100) | 0 (0) | 0 (0) | 0 (0) | 116 (99.1) | 0 (0) | 0 (0) | 1 (0.9) |
| Lower respiratory tract infection | 117 (99.2) | 0 (0) | 1 (0.8) | 0 (0) | 117 (99.6) | 0 (0) | 0 (0) | 0 (0) |
| Nephrolithiasis | 117 (99.2) | 0 (0) | 1 (0.8) | 0 (0) | 117 (99.6) | 0 (0) | 0 (0) | 0 (0) |

None of the Serious Adverse Events recorded were treatment-related

REFERENCES

1. Danthiir V, Hosking DE, Nettelbeck T, Vincent AD, Wilson C, O'Callaghan N, Calvaresi E, Clifton P, Wittert GA. An 18-mo randomized, double-blind, placebo-controlled trial of DHA-rich fish oil to prevent age-related cognitive decline in cognitively normal older adults. Am J Clin Nutr 2018;107(5):754-62.

2. Howe PR, Buckley JD, Murphy KJ, Pettman T, Milte C, Coates AM. Relationship between erythrocyte omega-3 content and obesity is gender dependent. Nutrients 2014;6(5):1850-60.

3. Milte CM, Coates AM, Buckley JD, Hill AM, Howe PR. Dose-dependent effects of docosahexaenoic acid-rich fish oil on erythrocyte docosahexaenoic acid and blood lipid levels. Br J Nutr 2008;99(5):1083-8.

4. Pipingas A, Cockerell R, Grima N, Sinclair A, Stough C, Scholey A, Myers S, Croft K, Sali A, Pase MP. Randomized controlled trial examining the effects of fish oil and multivitamin supplementation on the incorporation of n-3 and n-6 fatty acids into red blood cells. Nutrients 2014;6(5):1956-70.

5. Street SJ, Parletta N, Milte C, Sullivan K, Hills AP, Buckley J, Howe P. Interaction of erythrocyte eicosapentaenoic acid and physical activity predicts reduced risk of mild cognitive impairment. Aging Ment Health 2015;19(10):885-91.

6. Sullivan BL, Williams PG, Meyer BJ. Biomarker validation of a long-chain omega-3 polyunsaturated fatty acid food frequency questionnaire. Lipids 2006;41(9):845-50.

7. Harris WS, Pottala JV, Varvel SA, Borowski JJ, Ward JN, McConnell JP. Erythrocyte omega-3 fatty acids increase and linoleic acid decreases with age: observations from 160,000 patients. Prostaglandins Leukot Essent Fatty Acids 2013;88(4):257-63.

8. Walker RE, Jackson KH, Tintle NL, Shearer GC, Bernasconi A, Masson S, Latini R, Heydari B, Kwong RY, Flock M, et al. Predicting the effects of supplemental EPA and DHA on the omega-3 index. Am J Clin Nutr 2019;110(4):1034-40.

9. Swierk M, Williams PG, Wilcox J, Russell KG, Meyer BJ. Validation of an Australian electronic food frequency questionnaire to measure polyunsaturated fatty acid intake. Nutrition 2011;27(6):641-6.

10. Altman R, Asch E, Bloch D, Bole G, Borenstein D, Brandt K, Christy W, Cooke TD, Greenwald R, Hochberg M, et al. Development of criteria for the classification and reporting of osteoarthritis. Classification of osteoarthritis of the knee. Diagnostic and Therapeutic Criteria Committee of the American Rheumatism Association. Arthritis Rheum 1986;29(8):1039-49.

11. Kellgren JH, Lawrence JS. Radiological assessment of osteo-arthrosis. Ann Rheum Dis 1957;16(4):494-502.

12. Felson DT, Naimark A, Anderson J, Kazis L, Castelli W, Meenan RF. The prevalence of knee osteoarthritis in the elderly. The Framingham Osteoarthritis Study. Arthritis Rheum 1987;30(8):914-8.

13. Gossec L, Jordan JM, Mazzuca SA, Lam MA, Suarez-Almazor ME, Renner JB, Lopez-Olivo MA, Hawker G, Dougados M, Maillefert JF. Comparative evaluation of three semi-quantitative radiographic grading techniques for knee osteoarthritis in terms of validity and reproducibility in 1759 X-rays: report of the OARSI-OMERACT task force. Osteoarthritis Cartilage 2008;16(7):742-8.

14. Wright RW. Osteoarthritis Classification Scales: Interobserver Reliability and Arthroscopic Correlation. J Bone Joint Surg Am 2014;96(14):1145-51.

15. Bellamy N. WOMAC Osteoarthritis Index: user guide IX. Brisbane: Nicholas Bellamy; 2008. 78 p.

16. Harris PA, Taylor R, Minor BL, Elliott V, Fernandez M, O'Neal L, McLeod L, Delacqua G, Delacqua F, Kirby J, et al. The REDCap consortium: Building an international community of software platform partners. J Biomed Inform 2019;95:103208.

17. Harris PA, Taylor R, Thielke R, Payne J, Gonzalez N, Conde JG. Research electronic data capture (REDCap)--a metadata-driven methodology and workflow process for providing translational research informatics support. J Biomed Inform 2009;42(2):377-81.
